# A stochastic model for immunotherapy of cancer

**DOI:** 10.1038/srep24169

**Published:** 2016-04-11

**Authors:** Martina Baar, Loren Coquille, Hannah Mayer, Michael Hölzel, Meri Rogava, Thomas Tüting, Anton Bovier

**Affiliations:** 1 Institute for Applied Mathematics, Bonn University, Bonn, Germany; 2 Institute for Clinical Chemistry and Clinical Pharmacology, University Hospital, Bonn University, Bonn, Germany; 3 Laboratory of Experimental Dermatology, Department of Dermatology and Allergy, University Hospital, Bonn University, Bonn, Germany; 4 Department of Dermatology, University Hospital, Magdeburg University, Germany

## Abstract

We propose an extension of a standard stochastic individual-based model in population dynamics which broadens the range of biological applications. Our primary motivation is modelling of immunotherapy of malignant tumours. In this context the different actors, T-cells, cytokines or cancer cells, are modelled as single particles (individuals) in the stochastic system. The main expansions of the model are distinguishing cancer cells by phenotype and genotype, including environment-dependent phenotypic plasticity that does not affect the genotype, taking into account the effects of therapy and introducing a competition term which lowers the reproduction rate of an individual in addition to the usual term that increases its death rate. We illustrate the new setup by using it to model various phenomena arising in immunotherapy. Our aim is twofold: on the one hand, we show that the interplay of genetic mutations and phenotypic switches on different timescales as well as the occurrence of metastability phenomena raise new mathematical challenges. On the other hand, we argue why understanding purely stochastic events (which cannot be obtained with deterministic models) may help to understand the resistance of tumours to therapeutic approaches and may have non-trivial consequences on tumour treatment protocols. This is supported through numerical simulations.

Immunotherapy of cancer received a lot of attention in the medical as well as the mathematical modelling communities during the last decades^1^,^2^,^3^,^4^,^5^,^6^. Many different therapeutic approaches were developed and tested experimentally. As for the classical therapies such as chemo- and radiotherapy, *resistance* is an important issue also for immunotherapy: although a therapy leads to an initial phase of remission, very often a relapse occurs. The main driving forces for resistance are considered to be the genotypic and phenotypic heterogeneity of tumours, which may be enhanced during therapy, see^5^,^6^,^7^ and references therein. A tumour is a complex tissue which evolves in mutual influence with its environment^8^.

In this article, we consider the example of melanoma (tumours associated to skin cancer) under T-cell therapy. Our work is motivated by the experiments of Landsberg *et al*.^9^, which investigate melanoma in mice under *adoptive cell transfer* (ACT) therapy. This therapeutic approach involves the injection of T-cells which recognise a melanocyte-specific antigen and are able to kill differentiated types of melanoma cells. The therapy induces an inflammation and the melanoma cells react to this environmental change by switching their phenotype, i.e. by passing from a differentiated phenotype to a dedifferentiated one (neural-crest progenitor-like phenotype). The T-cells recognise the cancerous cells through the melanocytic antigens which are downregulated in the dedifferentiated types. Thus, they are not capable of killing these cancer cells, and a relapse is often observed. The phenotype switch is enhanced, if pro-inflammatory cytokines, called TNF-*α* (Tumour Necrosis Factor), are present. A second reason for the appearance of a relapse is that the T-cells become exhausted and are not working efficiently anymore. This problem was addressed by re-stimulation of the T-cells, but this led only to a delay in the occurrence of the relapse. Of course, other immune cells and cytokines are also present. However, according to the careful control experiments, their influence can be neglected in the context of the phenomena considered here.

Cell division is not required for switching, and switching is reversible. This means that the melanoma cells can recover their initial (differentiated) phenotype^9^. The switch is thus a purely phenotypic change which is not induced by a mutation. The state of the tumour is a mixture of differentiated and dedifferentiated cells. One possibility to avoid a relapse is to inject two types of T-cells (one specific to differentiated cells as above, and the other specific to dedifferentiated cells) as suggested also in^9^.

In this paper, we propose a quantitative mathematical model that can reproduce the phenomena observed in the experiments of 9, and which allows to simulate different therapy protocols, including some where several types of T-cells are used. The model we propose is an extension of the *individual-based* stochastic models for adaptive dynamics that were introduced in Metz *et al*.^10^ and developed and analysed by many authors in recent years (see e.g.^11^,^12^,^13^,^14^,^15^,^16^,^17^,^18^,^19^,^40^) to the setting of tumour growth under immunotherapy. Such models are also referred to as *agent-based* models or *particle systems*. Note that we use the term individual for T-cells, cytokines or cancer cells, in particular not for patients or mice.

These stochastic models describe the evolution of interacting cell populations, in which the relevant events for each individual (e.g. birth and death) occur randomly. It is well known that in the limit of large cell-populations, these models are approximated by deterministic kinetic rate models, which are widely used in the modelling of cell populations. However, these approximations are inaccurate and fail to account for important phenomena, if the numbers of some sub-populations become small. In such situations, random fluctuation may become highly significant and completely alter the long-term behaviour of the system. For example, in a phase of remission during therapy, the cancer and the T-cell populations drop to a low level and may die out due to fluctuations.

A number of (mostly deterministic) models have been proposed that describe the development of a tumour under treatment, focusing on different aspects. For example, a deterministic model for ACT therapy is presented in^3^. Stochastic approaches were used to understand certain aspects of tumour development, for example rate models^20^ or multi-type branching processes; see the book by Durrett^21^ or^22^,^23^,^24^. To our knowledge, however, it is a novel feature of our models to describe the coevolution of immune- and tumour cells taking into account both interactions and phenotypic plasticity. Our model can help understanding the interplay of therapy and resistance, in particular in the case of immunotherapy, and may be used to predict successful therapy protocols.

## Results

### The model

We now give a more detailed description of the models we consider. They contain three types of actors: First, *cancer cells* characterised by a genotype and a phenotype. These cells can divide (with or without mutation), die (due to age, competition or therapy) and switch their phenotype. We assume that the switch is inherited by the descendants of the switched cells. Second, *T-cells* that can divide, die and produce cytokines. Third, *cytokines* which can vanish and influence the switching of cancer cells.

**Figure 1:**
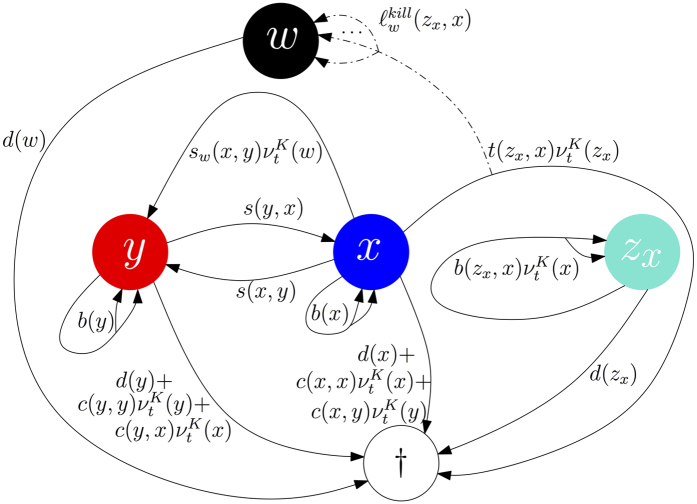
Dynamics of the process (without mutations) modelling the experiments described in^9^. Here, *x* denotes differentiated melanoma cells, *y* dedifferentiated melanoma cells, *z*_*x*_ T-cells and *w* TNF-*α*. At each arrow the rate for occurrence of the corresponding event is indicated (e.g. birth is illustrated with two arrowheads and death with an arrow directed to †).

The trait space, 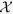, is a finite set of the form


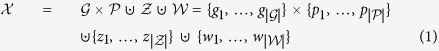


where 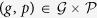 denotes a cancer cell with genotype *g* and phenotype *p*, 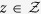 a T-cell of type *z* and 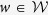 a cytokine of type *w*. We write |⋅| for the number of elements of a set and 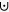 for disjoint unions of sets. Figure 1 provides a graphical representation of the transitions for a population with trait space 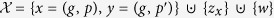.

A *population* at time 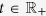 is represented by the measure


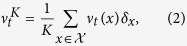


where *ν*_*t*_(*x*) is the number of individuals of type *x* at time *t* and *δ*_*x*_ denotes the Dirac measure at *x*. Here, *K* is a parameter that allows to scale the population size and is usually called *carrying-capacity* of the environment. The dynamics of the population is a continuous time Markov process, 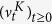 that is described by specifying the following *transition rates*:

Each cancer cell of type (*g, p*) is characterised by

- Natural birth and death rates: 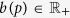 and 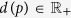.
- Competition kernels: 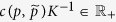 and 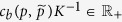, where the first term increases the death rate and the second term, called birth-reducing competition, lowers the birth rate of a cancer cell of phenotype *p* in presence of a cancer cell of phenotype 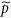. If the total birth rate is already at a level 0, then 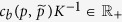 acts as an additional death rate.
- Therapy kernel: 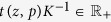 additional death rate of a cancer cell of phenotype *p* due to the presence of a T-cell of type *z*. In addition, 
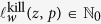
 cytokines of type *w* are deterministically produced at each killing event.
- Switch kernels: 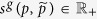 and 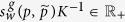 denote the natural and cytokine-induced switch kernels from a cancer cell of type (*g, p*) to one of type 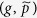.
- Mutation probability and law: *μ*_*g*_ ∈ [0, 1] denotes the probability that a birth event of a cancer cell of genotype *g* is a mutation. 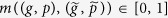 encodes the probability that, whenever a mutation occurs, a cancer cell of type (*g, p*) gives birth to a cancer cell of type 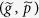. By definition *m*((*g, p*), (*g, p*)) = 0 and 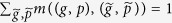.

Each T-cell of type *z* is characterised by

- Natural birth and death rates: 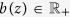 and 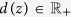.
- Reproduction kernel: 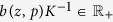 denotes the rate of reproduction of a T-cell with trait *z* in presence of a cancer cell of phenotype *p*. In addition, 
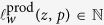
 cytokines of type *w* are deterministically produced at each reproduction event.

Each cytokine of type *w* is characterised by

- Natural death rate: 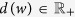.
- The molecules are produced when a cancer cell dies due to therapy or a T-cell reproduces.

Note that the relation between 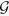 and 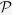 is encoded in the switch kernels. They specify which phenotypes are expressed by a given genotype and influence the proportions of the different phenotypes in a (dynamic) environment.

It is an important feature of stochastic models opposed to deterministic ones that populations can die out. There are two main reasons for the extinction of a population for finite *K*: first, the trajectory of the population size is transient and passes typically through a low minimum. In this case, random fluctuations can lead to extinction before the population reaches its equilibrium. Second, fluctuations around a finite equilibrium cause extinction of a population after a long enough time. The second case happens at much longer times scales than the first one. In both cases, the value of *K* plays a crucial role, since it determines the amplitude of the fluctuations and thus the probability of extinction.

It is well-known that the sequence of Markov processes, 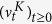, converges in the limit of large populations (*K* → ∞) to the solution of a system of quadratic, ordinary differential equations, see^25^. For this reason the deterministic system provides also (partial) information on the stochastic system. The deterministic system consists of a logistic part, a predator-prey relation between T-cells and cancer cells, a mutation and a switch part. The relevant mutations in the setup of cancer therapy are driver mutations and appear only rarely. In this case, more precisely, when the mutation probabilities, 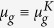, tend to zero as *K* → ∞, mutations are invisible in the deterministic limit. Due to the presence of the switches the analysis of the system is difficult. It is *not* a generalised Lotka-Volterra system of the form 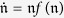, where *f* is linear in 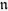. For precise mathematical descriptions, see the Supporting Information (SI).

In the last two sections of this article we show how to deal mathematically with rare mutations and their interactions with fast phenotypic switches or therapy.

### Therapy with T-cells of one specificity

In this section we present an example which *qualitatively* models the experiment of Landsberg *et al*.^9^, where melanoma escape ACT therapy by phenotypic plasticity in presence of TNF-*α*. Mutations are not considered here, since this was not investigated in the experiments. Let us denote by *x* := (*g, p*) the differentiated cancer cells, by *y* := (*g, p*′) the dedifferentiated cancer cells, by *z*_*x*_ the T-cells attacking only cells of type *x*, and by *w* the TNF-*α* proteins.

**Figure 2:**
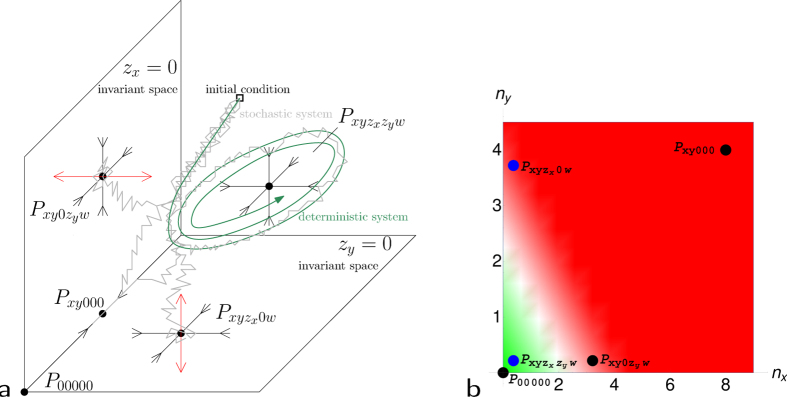
Qualitative description of the role of the fixed points. (**a**) Sketch of the invariant subspaces, stability of the fixed points, and schematic representation of the dynamics of the deterministic and the stochastic processes. (**b**) Projection of the fixed points onto 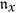 and 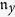. The green area contains the fixed points which correspond to cure or remission and the red area contains those describing relapses.

We start with describing the deterministic system and denote by 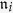 its solution for trait *i* ∈ {*x, y, z*_*x*_, *w*}. Let us explain here an example (parameters are given in the (SI)) with three fixed points, see the black dots on Fig. 2(b): *P*_0000_ where all populations sizes are zero,

*P*_*xy*00_ where the T-cells and TNF-*α* are absent and both melanoma populations are present and 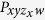 where all populations are present. 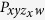 is the only stable fixed point and *P*_*xy*00_ is stable in the invariant subspace 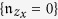 (i.e. if the T-cell population is zero). To highlight the qualitative features of the system, we choose parameters such that the minimum of the T-cell population during remission is low, and such that the equilibrium value of melanoma of type *x* in presence of T-cells is low, whereas equilibrium values of both melanoma types in absence of T-cells are high.

For initial conditions such that the number of differentiated melanoma cells, 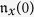, is large, the number of injected T-cells, 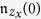, is small, and the numbers of dedifferentiated melanoma cells, 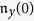, and TNF-*α* molecules, 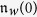, are small or equal to zero, the *deterministic* system is attracted to 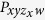: the T-cell population, 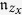, increases in presence of its target *x*, TNF-*α* is secreted, and the population of differentiated melanoma cells, 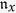, shrinks due to killing and TNF-*α* induced switching, whereas the population of dedifferentiated melanoma cells, 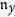, grows.

**Figure 3:**
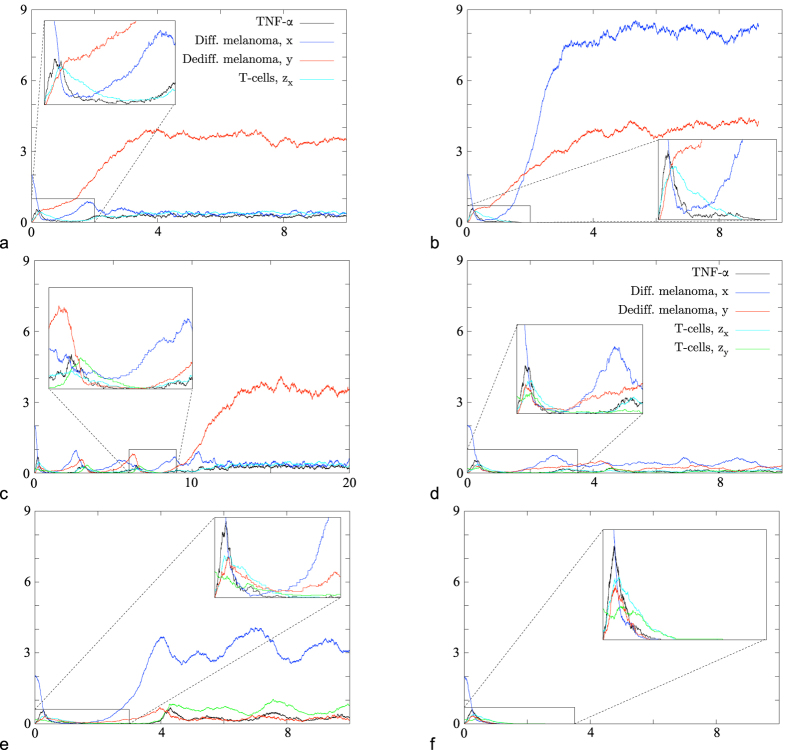
Simulations of the evolution of melanoma under T-cell therapy in the stochastic model for a fixed set of parameters. The graphs show the number of individuals divided by 200 versus time. Two scenarios are possible for therapy with T-cells of one specificity: (**a**) T-cells *z*_*x*_ survive and the system is attracted to 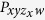, or (**b**) T-cells *z*_*x*_ die out and the system is attracted to *P*_*xy*00_. Three additional scenarios are possible for therapy with T-cells of two specificities: (**d**) T-cells *z*_*x*_ and *z*_*y*_ survive and the system stays close to 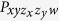, (**e**) T-cells *z*_*x*_ die out and the system is attracted to 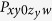, (**f**) the tumour is eradicated (corresponding to *P*_00000_). (**c**) Transition between cases (**a,d**).

For the stochastic system, several types of behaviour can occur with certain probabilities: either the trajectory stays close to that of the deterministic system and the system reaches a neighbourhood of the fixed point 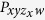 (Fig. 3(a)) or the T-cell population, *ν*^*K*^(*z*_*x*_), dies out and the system reaches a neighbourhood of *P*_*xy*00_ (Fig. 3(b)). In the latter case the TNF-*α* population, *ν*^*K*^(*w*), becomes extinct shortly after the extinction of the T-cells, *ν*^*K*^(*z*_*x*_), and the population of differentiated melanoma cells, *ν*^*K*^(*x*), can grow again. Moreover, TNF-*α* inducing the switch from *x* to *y* vanishes and we observe a relapse which consists mainly of differentiated cells. Depending on the choice of parameters (in particular switching, therapy or cross-competition), a variety of different behavior is possible.

### Therapy with T-cells of two specificities

A therapy can only be called successful if the whole tumour is eradicated or kept small for a long time. A natural idea is thus to inject two types of T-cells in future therapies as suggested in^9^. To model this scenario, we just need to add T-cells attacking the dedifferentiated cells as new actors to the setting described above. We denote them by *z*_*y*_. The system contains one more predator-prey term between *y* and *z*_*y*_ and can be found in the (SI). This adds two new fixed points (see the blue dots on Fig. 2(b)): 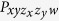 is the new stable fixed point with all non-zero populations, and 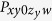 corresponds to the absence of the T-cell population of type *z*_*x*_. The invariant subspaces are now 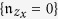, in which 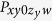 is stable, 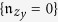, in which 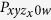 is stable and 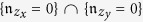, in which *P*_*xy*000_ is stable. Note that 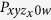, corresponding to 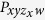 from the last section, is unstable in the enlarged space.

With the same initial conditions as before and 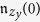 small but positive, the *deterministic* system is attracted to the stable fixed point 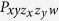: the T-cell population, 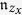, increases in presence of its target *x*, TNF-*α* is secreted, and the differentiated melanoma population shrinks due to killing and switching, the population of dedifferentiated melanoma grows, but is regulated and kept at a low level by the T-cells of type *z*_*y*_. Similarly, 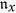 is regulated by 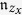. See (SI).

We choose the parameters such that the minima of the two types of T-cells during remission are low, so that they have a large enough probability to die out in the stochastic system. Since at the beginning of therapy no or only very few dedifferentiated melanoma cells are present, the population of T-cells of type *z*_*y*_ starts growing only later. In order to avoid their early extinction a higher initial amount of these T-cells can be injected. There are now five main different scenarios in the stochastic system (see Fig. 3). Either the T-cells of type *z*_*x*_ (e), or the T-cells of type *z*_*y*_, or both of them die out. The latter two cases are similar to Fig. 3(a,b) and thus only shown in the (SI). Also all populations can survive for some time fluctuating around their joint equilibrium (d). The fifth scenario is a cure, i.e. the extinction of the entire tumour due to the simultaneous attack of the two different T-cell types (f). T-cells and TNF-*α* vanish since they are not produced any more in the absence of their target. Of course, transitions between the different scenarios are also possible, e.g. the system could pass from Case (d) to (e) or (a) and then to (b), see Fig. 3(c). Furthermore, note that setting the switch from *x* to *y* to zero introduces an additional scenario: it is then possible that a relapse appears, which consists only of differentiated melanoma cells.

Starting from our choice of initial conditions, the deterministic system converges to 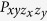, but the stochastic system can hit one of the invariant hyperplanes due to fluctuations, and is driven to different possible fixed points, see Fig. 2(a). The transitions between the different scenarios can be seen as a metastability phenomenon.

### Reproduction of experimental observations and prediction of the efficacy of the therapy

The parameters of the previous subsection are chosen ad hoc to highlight the influence of randomness and the possible behaviour of the system. Let us now show that our models are capable to reproduce the experimental data of Landsberg *et al*.^9^ *quantitiatvely*. The choice of parameters is explained in the (SI).

**Figure 4:**
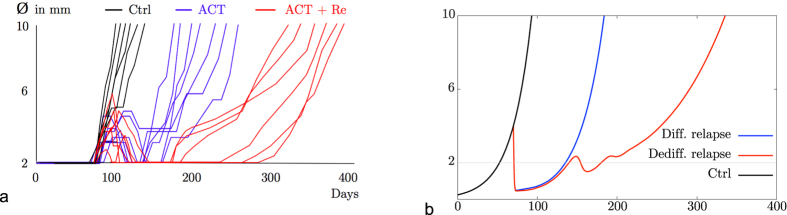
Comparison of experimental data obtained by Landsberg *et al*. with simulations for biologically reasonable parameters. The graphs show the diameter of the tumour measured in millimetres versus time in days after tumour initiation: (**a**) experimental data, (**b**) simulated data (*K* = 10^5^ and 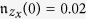).

Figure 4(a) shows the experimental data of 9 whereas Fig. 4(b) shows the results of our simulations. Each curve describes the evolution of the diameter of the tumour over time. In the stochastic system two situations can occur: first, the relapse consists mainly of differentiated melanoma cells and the tumour reaches its original size again after 90 days. This is the case if the T-cells die out. Second, the relapse consists mainly of dedifferentiated cells and the tumour reaches its original size again after roughly 190 days. This is the case if the T-cells survive the phase of remission, become active again and kill differentiated cancer cells. In the simulations the therapy with one type of T-cells pushes the tumour down to a microscopic level for 50 to 60 days, as in the experimental data. The curves marked ACT in the experimental data in Fig. 4(a) are matched by simulation data when the T-cells die out (Differentiated Relapse in Fig. 4(b)). In the experiments there might be T-cells which lose their function, e.g. due to exhaustion, and cannot kill the differentiated melanoma cells. This effect is to be seen as included in the death rate of T-cells in the model. They can be re-stimulated and become active again which is marked as ACT + Re in Fig. 4(a). Although our model does not include re-stimulation, the case of surviving T-cells in the simulations (Dediff. Relapse in Fig. 4(b)) can qualitatively be interpreted as the case of ACT + Re. Note that the scales of the axes are the same in both figures and that the experimental findings are met very well by the simulations. The simulated curves under treatment start at the beginning of the treatment and not at day zero.

As there is no data for the case of two T-cells, numerical simulations of such a therapy strategy should be seen as predictions. For the new T-cell population (of type *z*_*y*_) we choose the same parameters as for the first population (of type *z*_*x*_), just the target is different. The therapy seems to be very promising: almost all simulations show a cure for these parameters, only very few times a relapse occurs. Nevertheless, the behaviour of the system (e.g. the probability to end up in the different scenarios) depends strongly on the choice of certain parameters, as pointed out in the last two sections. In order to give a reliable prediction we need data to obtain safer estimates for the most important parameters, which seem to be the switching and therapy rates as well as initial values.

**Figure 5:**
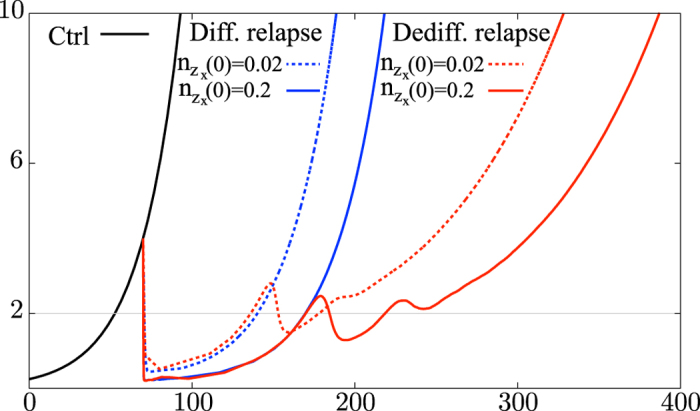
Simulations for different initial doses of T-cells 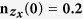 and 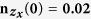.

The initial values play an important role for the success of a therapy. In the case of therapy with T-cells of one specificity, increasing the initial amount of T-cells has the following effect: the melanoma cells are killed faster, the population of differentiated melanoma cells reaches a lower minimum and as a consequence the T-cells pass through a lower and broader minimum. The probability that the T-cells die out increases, and a differentiated relapse is more likely than in the case of a smaller initial T-cell population. Moreover, the broadening of the minima causes a “delay” and both kind of relapses (consisting mainly of differentiated or dedifferentiated cells) appear later. But since the extinction of T-cells is more likely, the tumour may reach its original size earlier, see Fig. 5. For an initial value ten times as large as in Fig. 4(b) the probability of an eradication of the tumour is still very small. If the number of T-cells initially is half the number of tumour cells, the probability of a favourable outcome is much higher. But such a high amount of T-cells is unrealistic.

### Arrival of a mutant

In this section, we consider the case of rare mutations in large populations on a timescale such that a population reaches equilibrium before a new mutant appears (i.e. 
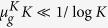
). The long-term behaviour of the standard models for adaptive dynamics is described in this limit by the Trait Substitution Sequence (see^10^,^15^) which is a Markov jump process on the trait space. A natural extension to the case where traits can coexist is the Polymorphic Evolution Sequence^26^. In the setting we consider here, several interesting new features arise that are so far only partially explored. One fundamental concept in the analysis of the stochastic population models is *invasion fitness*: for a given population in a stable equilibrium that populates a certain set of traits, say 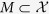, the invasion fitness *f* (*x, M*) is the growth rate of a population consisting of a single individual with trait 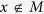 in the presence of the equilibrium population 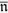 on *M*. Positive *f* (*x, M*) implies that a mutant with phenotype *x* has a positive probability (uniformly in *K*) to grow to a population of size of order *K*; negative invasion fitness implies that such a mutant population will die out with probability tending to one (as *K* → ∞). This notion needs to be generalised to the case when fast phenotypic switches are present. We sketch here how this can be done in the case of pure tumour evolutions, i.e. we ignore T-cells and TNF-*α*, for more details see (SI). The stochastic system can be approximated by a multi-type branching process (MTBP) until the mutant population dies out or reaches a size *εK* (for some small *ε* > 0). MTBPs have been analyzed e.g. in^27^,^28^,^29^,^30^. If we assume that the switch rates *s*(*p*_*i*_, *p*_*j*_) are the transition rates of an irreducible Markov chain (the simplest example is when *s*(*p*_*i*_, *p*_*j*_) > 0, for all *p*_*i*_ ≠ *p*_*j*_), then the behaviour of MTBP is classified in terms of its mean matrix *A*. It is well-known that the MTBP is supercritical if and only if the largest eigenvalue *λ*_1_ of *A* is strictly positive. In this case the mutant population will grow with positive probability to any desired population size before dying out. Thus *λ*_1_ is the appropriate generalization of the invasion fitness. The proportions **v** of the mutant population’s phenotypes at level *εK* can be deduced from limit theorems for MTBP which are explained in the (SI). Note that this notion of invasion fitness of course reduces to the standard one if there is only one mutant phenotype.

Once the mutant population has reached level *εK*, the behaviour of the process can be approximated by the solution of the corresponding deterministic system with initial value 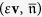. If this system is attracted to the same stable fixed point for all initial values in a neighbourhood of 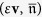, we are in the same situation as in^26^ and get a generalization of the Polymorphic Evolution Sequence. Observe that when the system has multiple attractors, and different points near the initial condition lie in different basins of attraction, the attractor approached by the system may be random for finite *K*. The characterization of the asymptotic behaviour of the system is needed to describe the final state of our stochastic process. This is in general a very difficult and complex problem, which is not doable analytically and will require numerical analysis.

**Figure 6:**
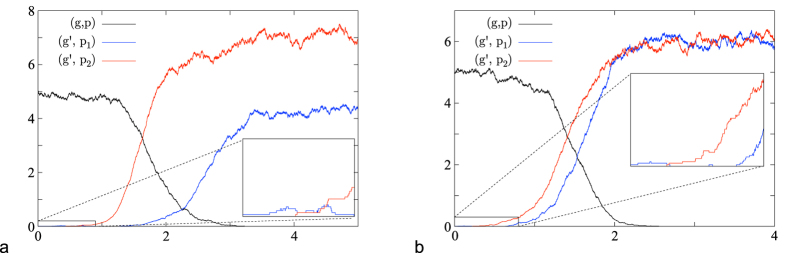
Simulations for rare mutations in combination with fast switching, where the number of individuals divided by 200 is plotted versus time. (**a**) The mutant phenotype *p*_2_ has a negative initial growth rate but can switch to *p*_1_ which has a positive one. The fitness of the genotype *g*′ is positive. (**b**) The fitness of the mutant genotype *g*′ is positive, although each phenotype has a negative initial growth rate. This is possible because an outgoing switch is a loss of a particle for a phenotype, but not for the whole genotype.

Figure 6 shows examples where in a population consisting only of type (*g, p*) a mutation to genotype *g*′ occurs. *g*′ is associated to two possible phenotypes *p*_1_ and *p*_2_. The parameters are given in the (SI).

### Interplay of mutation and therapy

In the previous section we considered the probability of invasion of a mutant when the resident population is at an equilibrium given by a stable fixed point. In the context of therapy, there are phases when populations shrink and regrow due to treatment and relapse phenomena. In the medical literature, there are frequent allusions to the possibility that such growth cycles may induce fixation of a “super-resistant mutant”, see e.g.^5^,^31^,^32^. It is important to understand whether and under what circumstances such effects may happen. Here we show an example where the appearance of a mutant genotype may indeed be enhanced under treatment. We consider *birth-reducing competition* (BRC) between tumour cells. In such a case, a large population at equilibrium may encounter fewer births and hence mutations, than a smaller population growing towards its equilibrium size.

For the sake of simplicity let the switch rates vanish. Consider a melanoma population (*g, p*) which is able to mutate to a fitter type of melanoma (*g*′, *p*′). If the competition is only of birth-reducing type, then the initial total mutation rate is 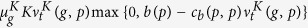. This is a quadratic function of 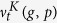. Without or before therapy, and before a mutant occurs, 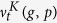 is close to the solution of the logistic deterministic system, which is attracted to the stable equilibrium 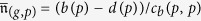. The mutation rate at 
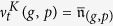
 is not maximal if *d*(*p*) is smaller than *b*(*p*)/2. Smaller populations can thus have a higher total mutation rate. More details are given in the (SI).

The interesting scaling of the mutation rate is 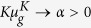 as *K* → ∞. In this case, there is a number of mutations of order one while the population grows by 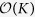 individuals. For lower mutation rates, no mutant can be expected before the population reaches its equilibrium, while for higher rates, mutations occur unrealistically fast. Since for 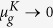 the mutation term does not appear in the deterministic system, the difference between BRC and usual competition is invisible. The effects described in this section are intrinsically stochastic and happen on timescales that diverge with *K*.

**Figure 7:**
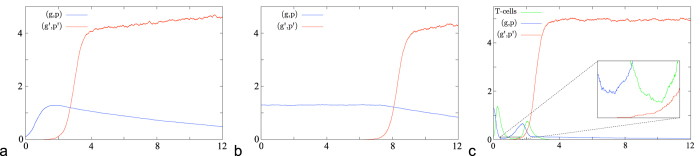
Simulations of mutation events in a population, where competition is acting via birth reduction. The number of individuals divided by 1000 is plotted versus time: Effect for an initial population which is small (**a**), or at equilibrium (**b**) or under therapy (**c**).

During therapy, a tumour which is close to equilibrium (similar to Fig. 7(b)) can shrink to a small size (similar to Fig. 7(a)): the introduction of T-cells in the system lowers the population size of melanoma, and the total mutation rate in the tumour population of type (*g, p*) can be larger during treatment, see Fig. 7(c). The simulations are obtained with parameters given in (SI). This means that treatment could lead to earlier mutations and thereby accelerate the evolution towards more aggressive tumour variants.

## Discussion

Therapy resistance is a major issue in the treatment of advanced stages of cancer. We have proposed a stochastic mathematical model that allows to simulate treatment scenarios and applied it to the specific case of immunotherapy of melanomas. Comparison to experimental data is so far promising. The models pose challenging new mathematical questions, in particular due to the interplay of fast phenotypic switches and rare driver mutations. First numerical results point to a significant effect of stochastic fluctuations in the success of therapies. More precise experimental data will be needed in the future to fit crucial model parameters. While our models describe the actions of individual cells and cytokines, they do not by far resolve the full complexity of the biological system. In particular, they do not reflect the spatial structure of the tumour and its microenvironment. Also, the distinction of only two phenotypes of the tumour cells is a simplification. The same is true for the interaction with other immune cells and cytokines. This reflects on the one hand the limitation due to available experimental data, on the other hand the use of a model of reduced complexity also makes numerical computations and theoretical understanding of the key phenomena feasible. The rates entering as model parameters therefore have to be understood as *effective* parameters, e.g. the death rate of T-cells accounts for their natural death as well as the exhaustion phenomenon. In principle it is possible to increase the resolution of the model; this, however, increases the number of parameters that need to be determined experimentally which would pose a major challenge. Already at the present stage, the model parameters are not known well enough and are adjusted to reproduce the experimental findings. Some parameters that it would be very useful to see measured precisely are:

- Birth and death rates of tumour cells, both in differentiated and dedifferentiated types. Currently these are estimated from the growth rate of the tumour, but this yields only the difference of these rates;
- Killing rates of T-cells, both of the differentiated and the dedifferentiated tumour cells;
- Rates of phenotypic switches, both in the absence and the presence of TNF-*α*;
- Death rates of T-cells and their expansion rates when interacting with tumour cells.

Nevertheless, we see the proposed model as a promising tool to assist the development of improved treatment protocols. Simulations may guide the choice of experiments such that the number of necessary experiments can be reduced. The obvious strength of our approach is to model reciprocal interactions and phenotypic state transitions of tumour and immune cells in a heterogeneous and dynamic microenvironment in the context of therapeutic perturbations.

The clinical importance of phenotypic coevolution in response to therapy has been recently documented in patients’ samples from melanomas acquiring resistance to MAPK inhibitors^33^. Adaptive activation of bypass survival pathways in melanoma cells was accompanied by the induction of an exhausted phenotype of cytolytic T cells. This has important implications for the combinatorial use of cancer immunotherapy (checkpoint inhibitors like anti PD-1) with respect to scheduling. We envision that our mathematical approach will help to integrate such patient omics data with experimental findings to guide novel strategies. Of note and similar to our previous study, dedifferentiation of melanoma cells was identified as a major mechanism of escape from MAPK inhibitors^34^,^35^. We recently dissected the molecular circuitries that control melanoma cell states and showed how melanoma dedifferentiation governs the composition of the immune cell compartment through a cytokine-based crosstalk in the microenvironment^36^,^37^. Hence, malignant melanoma is a paradigm for a phenotypic heterogeneous tumour and a future goal is to incorporate this increasing knowledge of melanoma cell plasticity into our method to refine its capability to model complex interactions with immune cells.

Importantly, phenotypic plasticity in response to therapy is a widespread phenomenon and non-small cell lung cancer (NSCLC) represents a prominent example. A subset of NSCLCs harbour activating mutations in the epidermal-growth factor receptor EGFR and respective small molecule inhibitors (EGFRi) are potent first line cancer drugs for this NSCLC subtype. However, tumours invariably relapse and genetic selection of subclones with the second site resistance mutation EGFRT790M is the major event. A substantial number of relapse tumours show remarkable transitions from an NSCLC adenocarcinoma to a neuroendocrine-related small cell lung cancer (SCLC) phenotype^38^. Given the recent success of immune checkpoint inhibitors in NSCLC^39^, it will be of clinical interest to investigate the phenotypic coevolution of immune cells in the context of NSCLC-SCLC lineage transitions. Again, our mathematical approach could represent a valuable tool to support this research. Finally, our results suggest that stochastic events play an unanticipated critical role in the dynamic evolution of tumours and the emergence of therapy resistance that requires further experimental and clinical investigation.

## Methods

Our simulations are based on a Gillespie algorithm. All possible events, together with their occurrence times, are simulated, i.e. each jump of the stochastic process described in the section “The model” is contained in the output. See the pseudo-code in (SI).

## Additional Information

**How to cite this article**: Baar, M. *et al*. A stochastic model for immunotherapy of cancer. *Sci. Rep.* **6**, 24169; doi: 10.1038/srep24169 (2016).

## Supplementary Material

Supplementary Information
