# Supplementary material for: A stochastic model for immunotherapy of cancer
Source: Sci Rep. 2016 Apr 11;6:24169. doi: 10.1038/srep24169 (PMC4827069; doi:10.1038/srep24169)
Supplement: Supplementary Information [file srep24169-s1.pdf]

SUPPLEMENTARY INFORMATION

A stochastic model for immunotherapy of cancer

Martina Baar†[1], Loren Coquille†*[1], Hannah Mayer†[1], Michael Hölzel[2], Meri Rogava[3], Thomas Tüting[3,4] and Anton Bovier [1]

[1]Institute for Applied Mathematics, Bonn University
[2]Institute for Clinical Chemistry and Clinical Pharmacology, University Hospital, Bonn University
[3]Laboratory of Experimental Dermatology, Department of Dermatology and Allergy, University Hospital, Bonn University
[4] Department of Dermatology, University Hospital, Magdeburg University
† Equal contribution
* Corresponding author

February 9, 2016

1. Mathematical description of the model

In this section we provide mathematical details of the stochastic individual-based model for immunotherapy of cancer that we introduced in the main text. Let $\mathcal{M}^K(\mathcal{X}) \equiv \left\{\frac{1}{K}\sum_{i=1}^n \delta_{x_i} : n \in \mathbb{N}, x_1, \ldots, x_n \in \mathcal{X}\right\}$, i.e. the set of finite point measures on $\mathcal{X}$ rescaled by K, where

$$\mathcal{X} = \mathcal{G} \times \mathcal{P} \cup \mathcal{Z} \cup \mathcal{W} = \left\{g_1, \ldots, g_{|\mathcal{G}|}\right\} \times \left\{p_1, \ldots, p_{|\mathcal{P}|}\right\} \cup \left\{z_1, \ldots, z_{|\mathcal{Z}|}\right\} \cup \left\{w_1, \ldots, w_{|\mathcal{W}|}\right\} \tag{1.1}$$

is the trait space. Then, for each $K \in \mathbb{N}$, the dynamics of the $\mathcal{M}^K(\mathcal{X})$-valued Markov process, $(\nu_t^K)_{t\geq 0}$, describing the evolution of the population at each time t, can be summarised as follows:

At the beginning the population is characterized by a given measure $\nu_0^K \in \mathcal{M}^K(\mathcal{X})$. Each individual present at time t has several exponential clocks with intensities depending on its trait $x \in \mathcal{X}$ and on the current state of the system: (We use below the shorthand notation $\nu_t^K(p) = \sum_{g\in\mathcal{G}} \nu_t^K(g,p)$ and $\lfloor\cdot\rfloor_+$ denotes the positive and $\lfloor\cdot\rfloor_-$ the negative part of the argument.)

(1) Each present cancer cell of trait $(g,p) \in \mathcal{G} \times \mathcal{P}$ has

- a *clonal reproduction* clock with rate $(1-\mu_g)\left\lfloor b(p) - \sum_{\tilde{p}\in\mathcal{P}} c_b(p,\tilde{p})\nu_t^K(\tilde{p})\right\rfloor_+$.

  Whenever this one rings, an additional cancer cell of the same trait (g,p) appears.

- a *mutant reproduction* clock with rate $\mu_g \left\lfloor b(p) - \sum_{\tilde{p}\in\mathcal{P}} c_b(p,\tilde{p})\nu_t^K(\tilde{p})\right\rfloor_+$.

  Whenever this one rings, a cancer cell of trait $(\tilde{g},\tilde{p})$ appears according to the kernel $m((g,p),(\tilde{g},\tilde{p}))$.

- a *natural mortality* clock with rate $d(p) + \sum_{\tilde{p}\in\mathcal{P}} c(\tilde{p},p)\nu_t^K(\tilde{p}) + \left\lfloor b(p) - \sum_{\tilde{p}\in\mathcal{P}} c_b(p,\tilde{p})\nu_t^K(\tilde{p})\right\rfloor_-$.

  Whenever this one rings, the cancer cell disappears.

- a *therapy mortality* clock of rate $\sum_{z\in\mathcal{Z}} t(z,p)\nu_t^K(z)$.

  Whenever this one rings, the cancer cell disappears and $\ell_w^{\text{kill}}(z,p)$ cytokines of type w appear according to the weights $t(z,p)\nu_t^K(z)$.

- a *natural and cytokine-induced switch* clock with rate $\sum_{\tilde{p}\in\mathcal{P}}(s^g(p,\tilde{p}) + \sum_{w\in\mathcal{W}} s_w^g(p,\tilde{p})\nu_t^K(w))$.

  Whenever this one rings, this cancer cell disappears and a new cancer cell of trait $(g,\tilde{p})$ appears according to the weights $s^g(p,\tilde{p}) + \sum_{w\in\mathcal{W}} s_w^g(p,\tilde{p})\nu_t^K(w)$.

(2) Each present T-cell of trait $z \in \mathcal{Z}$ has

- a *natural birth* clock with rate $b(z)$
- a *natural mortality* clock with rate $d(z)$
- a *reproduction* clock with rate $\sum_{p\in\mathcal{P}} b(z,p)\nu_t^K(p)$.

Whenever the reproduction clock of a T-cell rings, a additional T-cell with the same trait z and $\ell_w^{\text{prod}}(z,p)$

cytokines of type w appear according to the weights $b(z,p)\nu_t^K(p)$.

(3) Each present cytokine has

- a *mortality* clock with rate $\quad d(w)$.

Moreover, an amount of $\ell_w^{\text{kill}}(z,p)$ particles (of trait w) are produced every time a T-cell of trait z kills a cancer cell of phenotype p, and a number of $\ell_w^{\text{prod}}(z,p)$ particles are produced every time a T-cell of trait z is produced in the presence of a cancer cell of phenotype p.

The measure-valued process $(\nu_t^K)_{t\geq 0}$ is a Markov process whose law is characterized by its infinitesimal generator L^K which captures the dynamics described above (cf. [1] Chapter 11 and [2]). The generator acts on bounded measurable functions ϕ from $\mathcal{M}^K$ into $\mathbb{R}$, for all $\eta \in \mathcal{M}^K$ by

$$
\begin{aligned}
\left(L^K\phi\right)(\eta) = & \sum_{(g,p)\in\mathcal{G}\times\mathcal{P}} \left(\phi\left(\eta+\tfrac{\delta_{(g,p)}}{K}\right)-\phi(\eta)\right)(1-\mu_g)\left\lfloor b(p)-\sum_{\tilde p\in\mathcal{P}} c_b(p,\tilde p)\eta(\tilde p)\right\rfloor_+ K\eta(g,p) \\
& + \sum_{(g,p)\in\mathcal{G}\times\mathcal{P}} \left(\phi\left(\eta-\tfrac{\delta_{(g,p)}}{K}\right)-\phi(\eta)\right) \\
& \qquad \times\left(d(p)+\sum_{\tilde p\in\mathcal{P}} c(p,\tilde p)\eta(\tilde p)+\left\lfloor b(p)-\sum_{\tilde p\in\mathcal{P}} c_b(p,\tilde p)\eta(\tilde p)\right\rfloor_-\right)K\eta(g,p) \\
& + \sum_{(g,p)\in\mathcal{G}\times\mathcal{P}}\sum_{z\in\mathcal{Z}} \left(\phi\left(\eta-\tfrac{\delta_{(g,p)}}{K}+\textstyle\sum_{w\in\mathcal{W}}\ell_w^{\text{kill}}(z,p)\tfrac{\delta_w}{K}\right)-\phi(\eta)\right) t(z,p)\eta(z)K\eta(g,p) \\
& + \sum_{(g,p)\in\mathcal{G}\times\mathcal{P}}\sum_{\tilde p\in\mathcal{P}} \left(\phi\left(\eta+\tfrac{\delta_{(g,\tilde p)}}{K}-\tfrac{\delta_{(g,p)}}{K}\right)-\phi(\eta)\right) \\
& \qquad \times\left(s^g(p,\tilde p)+\textstyle\sum_{w\in\mathcal{W}} s_w^g(p,\tilde p)\eta(w)\right)K\eta(g,p) \\
& + \sum_{z\in\mathcal{Z}}\sum_{p\in\mathcal{P}} \left(\phi\left(\eta+\tfrac{\delta_z}{K}+\textstyle\sum_{w\in\mathcal{W}}\ell_w^{\text{prod}}(z,p)\tfrac{\delta_w}{K}\right)-\phi(\eta)\right)\left(b(z,p)\eta(p)\right)K\eta(z) \\
& + \sum_{z\in\mathcal{Z}} \left(\phi\left(\eta+\tfrac{\delta_z}{K}\right)-\phi(\eta)\right) b(z)K\eta(z) \\
& + \sum_{z\in\mathcal{Z}} \left(\phi\left(\eta-\tfrac{\delta_z}{K}\right)-\phi(\eta)\right) d(z)K\eta(z) \\
& + \sum_{w\in\mathcal{W}} \left(\phi\left(\eta-\tfrac{\delta_w}{K}\right)-\phi(\eta)\right) d(w)K\eta(w) \\
& + \sum_{(\tilde g,\tilde p)\in\mathcal{G}\times\mathcal{P}}\sum_{(g,p)\in\mathcal{G}\times\mathcal{P}} \left(\phi\left(\eta+\tfrac{\delta_{(\tilde g,\tilde p)}}{K}\right)-\phi(\eta)\right) \\
& \qquad \times\mu_g m((g,p),(\tilde g,\tilde p))\left\lfloor b(p)-\sum_{p'\in\mathcal{P}} c_b(p,p')\eta(p')\right\rfloor_+ K\eta(g,p)
\end{aligned} \tag{1.2}
$$

where we use the short hand notation $\eta(g,p)\equiv\eta((g,p))$ and $\eta(p)\equiv\sum_{g\in\mathcal{G}}\eta(g,p)$.

There is a natural way to jointly construct the processes for different values of K, see [1]. The sequence of rescaled processes $((\nu_t^K)_{t\geq 0})_K$ converges almost surely as K tends to infinity to the solution of a quadratic system of ODEs, as stated in the following proposition, which can be seen as a law of large numbers.

Note that the population can be represented as a vector $V_K(t) := (\nu_t^K(x))_{x\in\mathcal{X}}$ of dimension $d=|\mathcal{G}|\cdot|\mathcal{P}|+|\mathcal{Z}|+|\mathcal{W}|$. We choose to present the population as a measure in the definition of the model, since this is more convenient for large trait spaces.

Proposition 1. *Suppose that the initial conditions converge almost surely to a deterministic limit, i.e.* $\lim_{K\to\infty} V_K(0) = v(0)$. *Then, for each* $T \in \mathbb{R}_+$ *the sequence of rescaled processes* $(V_K(t))_{0\le t\le T}$ *converges almost surely as* $K \to \infty$ *to the d-dimensional deterministic process which is the unique solution to the following quadratic dynamical system:*
For all $(g,p) \in \mathcal{G} \times \mathcal{P}$,

$$\begin{aligned}\frac{\mathrm{d}\mathfrak{n}_{(g,p)}}{\mathrm{d}t} &= \mathfrak{n}_{(g,p)} \Bigg((1-\mu_g) \Bigg\lfloor b(p) - \sum_{(\tilde g,\tilde p)\in\mathcal{G}\times\mathcal{P}} c_b(p,\tilde p)\mathfrak{n}_{(\tilde g,\tilde p)} \Bigg\rfloor_+ \\ &\qquad - \Bigg\lfloor b(p) - \sum_{(\tilde g,\tilde p)\in\mathcal{G}\times\mathcal{P}} c_b(p,\tilde p)\mathfrak{n}_{(\tilde g,\tilde p)} \Bigg\rfloor_- - d(p) - \sum_{(\tilde g,\tilde p)\in\mathcal{G}\times\mathcal{P}} c(p,\tilde p)\mathfrak{n}_{(\tilde g,\tilde p)} \\ &\qquad - \sum_{z\in\mathcal{Z}} t(z,p)\mathfrak{n}_z - \sum_{\tilde p\in\mathcal{P}} \Big(s^g(p,\tilde p) + \sum_{w\in\mathcal{W}} s^g_w(p,\tilde p)\mathfrak{n}_w \Big) \Bigg) \\ &\quad + \sum_{\tilde p\in\mathcal{P}} \mathfrak{n}_{(g,\tilde p)} \Big(s^g(\tilde p,p) + \sum_{w\in\mathcal{W}} s^g_w(\tilde p,p)\mathfrak{n}_w \Big) \\ &\quad + \sum_{(\tilde g,\tilde p)\in\mathcal{G}\times\mathcal{P}} \mathfrak{n}_{(\tilde g,\tilde p)} \Bigg(\mu_{\tilde g}\, m((\tilde g,\tilde p),(g,p)) \Bigg\lfloor b(\tilde p) - \sum_{(g',p')\in\mathcal{G}\times\mathcal{P}} c_b(\tilde p,p')\mathfrak{n}_{(g',p')} \Bigg\rfloor_+ \Bigg),\end{aligned}$$

for all $z \in \mathcal{Z}$,

$$\frac{\mathrm{d}\mathfrak{n}_z}{\mathrm{d}t} = \mathfrak{n}_z \Bigg(b(z) - d(z) + \sum_{(g,p)\in\mathcal{G}\times\mathcal{P}} b(z,p)\mathfrak{n}_{(g,p)} \Bigg), \tag{1.3}$$

for all $w \in \mathcal{W}$,

$$\frac{\mathrm{d}\mathfrak{n}_w}{\mathrm{d}t} = -\mathfrak{n}_w d(w) + \sum_{(g,p)\in\mathcal{G}\times\mathcal{P}} \mathfrak{n}_{(g,p)} \sum_{z\in\mathcal{Z}} \Big(\ell^{\text{kill}}_w(z,p)\, t(z,p) + \ell^{\text{prod}}_w(z,p)\, b(z,p) \Big) \mathfrak{n}_z.$$

More precisely, $\mathbb{P}\left(\lim_{K\to\infty} \sup_{0\le t\le T} |V_K(t) - \mathfrak{n}(t)| = 0\right) = 1$, *where* $\mathfrak{n}(t)$ *denotes the solution to Equations* (1.3) *with initial condition* $v(0)$.

This result follows from Theorem 2.1 in Chapter 11 of [1].

2. Deterministic systems and parameter choice for the examples

2.1. **Therapy with T-cells of one specificity.** Recall that we denote by $x := (g,p)$ the differentiated cancer cells, by $y := (g,p')$ the dedifferentiated cancer cells, by z_x the T-cells attacking only cells of type x, and by w the TNF-α proteins. The reduced deterministic system corresponding to the example describing the experiments is the solution to the following system of four differential equations:

$$\begin{aligned}\dot{\mathfrak{n}}_x &= \mathfrak{n}_x\big(b(x) - d(x) - c(x,x)\mathfrak{n}_x - c(x,y)\mathfrak{n}_y - s(x,y) - s_w(x,y)\mathfrak{n}_w - t(z_x,x)\mathfrak{n}_{z_x}\big) + s(y,x)\mathfrak{n}_y \\ \dot{\mathfrak{n}}_y &= \mathfrak{n}_y\big(b(y) - d(y) - c(y,y)\mathfrak{n}_y - c(y,x)\mathfrak{n}_x - s(y,x)\big) + s(x,y)\mathfrak{n}_x + s_w(x,y)\mathfrak{n}_w\mathfrak{n}_x \\ \dot{\mathfrak{n}}_{z_x} &= \mathfrak{n}_{z_x}(b(z_x,x)\mathfrak{n}_x - d(z_x)) \\ \dot{\mathfrak{n}}_w &= -\mathfrak{n}_w d(w) + (\ell^{\text{kill}}_w(z_x,x)\, t(z_x,x) + \ell^{\text{prod}}_w(z_x,x)\, b(z_x,x))\mathfrak{n}_x\mathfrak{n}_{z_x}\end{aligned} \tag{2.1}$$

The parameters used in the simulations for the qualitative example are:

$$\begin{array}{llll} b(x) = 3 & b(y) = 3 & b(z_x,x) = 8 & \ell^{\text{kill}}_w(z_x,x) = 1 \\ d(x) = 1 & d(y) = 1 & t(z_x,x) = 28 & \ell^{\text{prod}}_w(z_x,x) = 0 \\ c(x,x) = 0.3 & c(y,x) = 0 & d(z_x) = 3 & d(w) = 15 \\ c(x,y) = 0 & c(y,y) = 0.3 & & s_w(x,y) = 4 \\ s(x,y) = 0.1 & s(y,x) = 1 & & \end{array} \tag{2.2}$$

the scaling parameter K and the initial conditions are:

$$K = 200, \quad (\mathfrak{n}_x, \mathfrak{n}_y, \mathfrak{n}_{z_x}, \mathfrak{n}_w)(0) = (2, 0, 0, 0.05, 0) \tag{2.3}$$

The solution to the deterministic system (2.1) with parameters (2.2) and initial conditions (2.3) can be seen on Figure 1 (A).

2.2. **Therapy with T-cells of two specificities.** Recall that we denote by z_y the T-cells attacking dedifferentiated melanoma. In the case where two types of T-cells are used, the deterministic system consists of the following five equations:

$$\begin{aligned}
\dot{\mathfrak{n}}_x &= \mathfrak{n}_x\Big(b(x) - d(x) - c(x,x)\mathfrak{n}_x - c(x,y)\mathfrak{n}_y - s_w(x,y)\mathfrak{n}_w - s(x,y) - t(z_x,x)\mathfrak{n}_{z_x}\Big) + s(y,x)\mathfrak{n}_y \\
\dot{\mathfrak{n}}_y &= \mathfrak{n}_y\Big(b(y) - d(y) - c(y,y)\mathfrak{n}_y - c(y,x)\mathfrak{n}_x - s(y,x) - t(z_y,y)\mathfrak{n}_{z_y}\Big) + \big(s_w(x,y)\mathfrak{n}_w + s(x,y)\big)\mathfrak{n}_x \\
\dot{\mathfrak{n}}_{z_x} &= \mathfrak{n}_{z_x}(b(z_x,x)\mathfrak{n}_x - d(z_x)) \\
\dot{\mathfrak{n}}_{z_y} &= \mathfrak{n}_{z_y}(b(z_y,y)\mathfrak{n}_y - d(z_y)) \\
\dot{\mathfrak{n}}_w &= -\mathfrak{n}_w d(w) + (\ell_w^{\text{kill}}(z_x,x)\,t(z_x,x) + \ell_w^{\text{prod}}(z_x,x)\,b(z_x,x))\mathfrak{n}_x\mathfrak{n}_{z_x} \\
&\qquad + (\ell_w^{\text{kill}}(z_y,y)\,t(z_y,y) + \ell_w^{\text{prod}}(z_y,y)\,b(z_y,y))\mathfrak{n}_y\mathfrak{n}_{z_y}
\end{aligned} \tag{2.4}$$

In addition to parameters (2.2), we use the following ones:

$$\begin{array}{lll} t(z_y,y) = 28 & \ell_w^{\text{kill}}(z_y,y) = 1 & d(z_y) = 3 \\ b(z_y,y) = 14 & \ell_w^{\text{prod}}(z_y,y) = 0 & \end{array} \tag{2.5}$$

the scaling parameter K and the initial conditions are:

$$K = 200, \quad (\mathfrak{n}_x, \mathfrak{n}_y, \mathfrak{n}_{z_x}, \mathfrak{n}_{z_y}, \mathfrak{n}_w)(0) = (2, 0, 0, 0.05, 0.2, 0) \tag{2.6}$$

The solution to the deterministic system (2.4) with parameters (2.2) and (2.5) and initial conditions (2.6) can be seen on Figure 1 (B). Initial conditions with only T-cells of type z_y or without any T-cells can be seen on Figure 1 (C) and (D). Additional simulations mentioned in the main part are available in Figure 2.

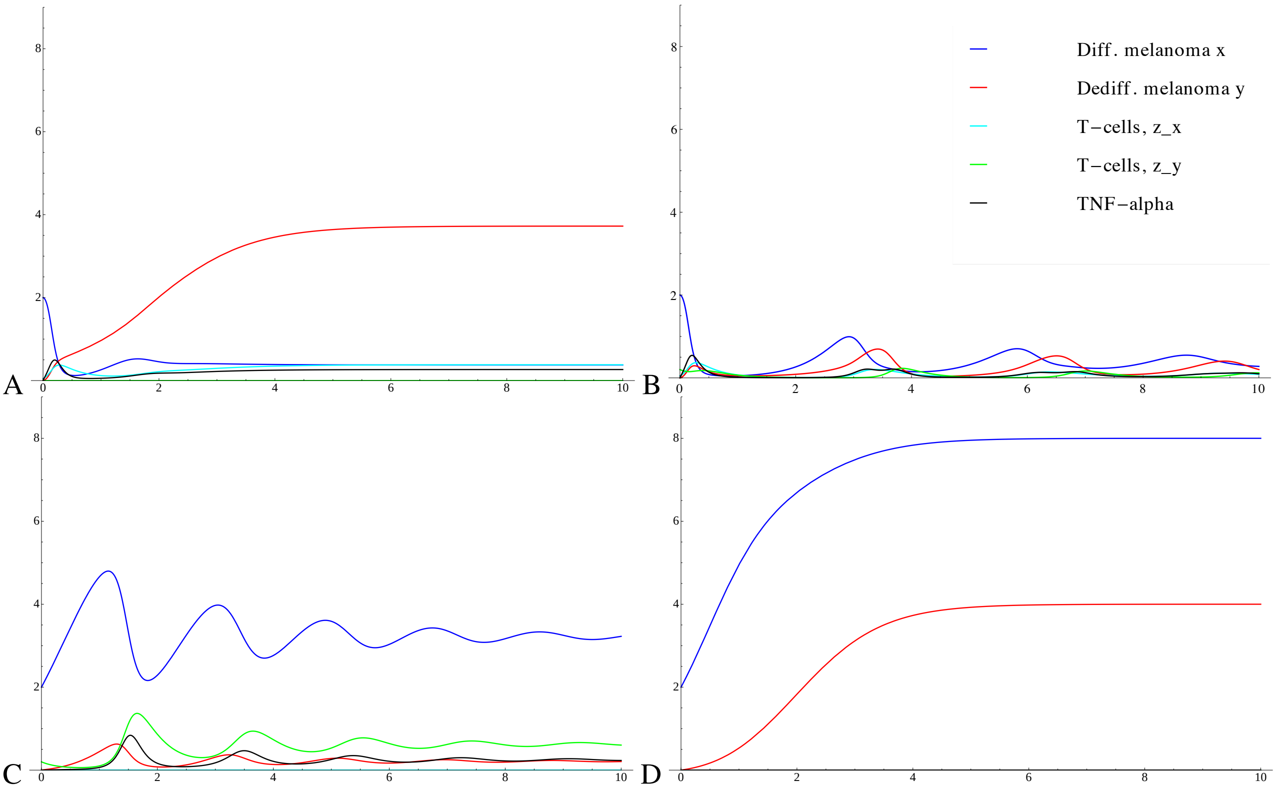

FIGURE 1. Solutions to the deterministic system (2.4) with numerical parameters (2.2) and (2.5) and the following initial conditions $\mathfrak{n}(0) := (\mathfrak{n}_x, \mathfrak{n}_y, \mathfrak{n}_{z_x}, \mathfrak{n}_{z_y}, \mathfrak{n}_w)(0)$:
(A) $\mathfrak{n}(0) = (2, 0, 0.05, 0, 0)$, the system is attracted to the fixed point P_{xyz_x0w},
(B) $\mathfrak{n}(0) = (2, 0, 0.05, 0.2, 0)$, the system is attracted to the fixed point $P_{xyz_xz_yw}$,
(C) $\mathfrak{n}(0) = (2, 0, 0, 0.2, 0)$, the system is attracted to the fixed point P_{xy0z_yw},
(D) $\mathfrak{n}(0) = (2, 0, 0, 0, 0)$, the system is attracted to the fixed point P_{xy000}.

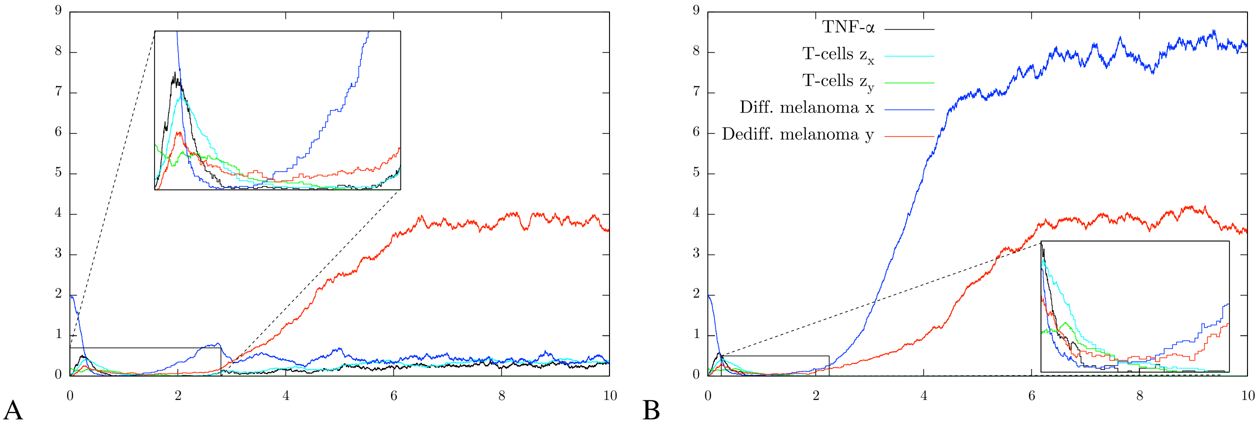

FIGURE 2. Therapy with two T-cell types. (A) T-cells z_y die out and the system is attracted to P_{xyz_x0w}, (B) Both T-cell types z_x and z_y die out and the system is attracted to P_{xy000}.

2.3. **Physiologically reasonable parameters.** We explain here how we choose the biological parameters. Some parameters can be estimated from the experimental data. Recall that the subject of [3] is to investigate the behaviour of melanoma under T-cell therapy in mice. Without therapy the tumour undergoes only natural birth, death and switch events.

- *Choice of birth and death rates:* We assume that the number of cells in the tumour is described by
$$N_t \approx N_0 \exp(rt), \tag{2.7}$$
where N_t denotes the number of cells at time t, N_0 the initial population size and r the overall growth rate. Note that the estimate of the growth rate is independent of the initial value. Fig. 4 (A) in the main part shows that the tumour needs roughly 50 days (without therapy) to grow from 2 mm diameter to 10 mm diameter. Since the structure of a melanoma is 3-dimensional this corresponds roughly to $N_{50} = 125N_0$ which implies $r = 0.1$. Unfortunately, no data that allow to estimate the ratio of birth and death events are provided. As long as mutations are not considered this should not have a big impact and we chose $b = 0.12$ and $d = 0.02$ for the differentiated as well as the dedifferentiated cells. Landsberg et al. observed that the growth kinetics appear to be the same for both cell types, see Supplementary Figure 11 in [3].

- *Choice of the competition:* We assume that the competition has a very little effect here because the tumour grows exponentially in the observed time frame and does not come close to its equilibrium. We choose the competition between melanoma cells of the same type as $c(x, x) = c(y, y) = 0.00005$ and between different types of melanoma cells as $c(x, y) = c(y, x) = 0.00002$. The values are not set to 0 since the melanoma can grow only up to a finite size.

- *Choice of the switch parameters:* We can now estimate (roughly) the switching parameters by using the data of Supplementary Figure 9e in [3]. In this experiment where cell division is inhibited, we can set $b = 0$. Furthermore, the amount of TNF-α is constant and we set here $\mathfrak{n}_w = 2$. Thus, the dynamics of the melanoma populations is described by
$$\begin{aligned} \dot{\mathfrak{n}}_x &= \mathfrak{n}_x\big(-d(x) - c(x,x)\mathfrak{n}_x - c(x,y)\mathfrak{n}_y - 2s_w(x,y) - s(x,y)\big) + s(y,x)\mathfrak{n}_y \\ \dot{\mathfrak{n}}_y &= \mathfrak{n}_y\big(-d(y) - c(y,y)\mathfrak{n}_y - c(y,x)\mathfrak{n}_x - s(y,x)\big) + \big(2s_w(x,y) + s(x,y)\big)\,\mathfrak{n}_x \end{aligned} \tag{2.8}$$
At the beginning of their observations the switch is very slow and speeds up after the first 24 hours. We assume that there is a delay until the reaction really starts and thus we choose the proportions at day 1 ($\mathfrak{n}_x = 0.81$ and $\mathfrak{n}_y = 0.19$) as initial data and choose switching parameters such that roughly the concentrations at day 2 ($\mathfrak{n}_x = 0.45$ and $\mathfrak{n}_y = 0.54$) and 3 ($\mathfrak{n}_x = 0.24$ and $\mathfrak{n}_y = 0.72$) are reached as shown in Figure 3. Thereby we obtain $s(x, y) = 0.0008$, $s(y, x) = 0.065$ and $s_w(x, y) = 0.33$. Note that the experiments we refer to provide only in vitro data and it is not clear if the in vivo situation is similar.

- *Choice of parameters concerning T-cells:* It remains to characterize the T-cells. Their natural birth rate is set to 0 since they are transferred by adoptive cell transfer and not produced by the mice themselves and do not proliferate in absence of targets. We assume that they have a relatively high birth

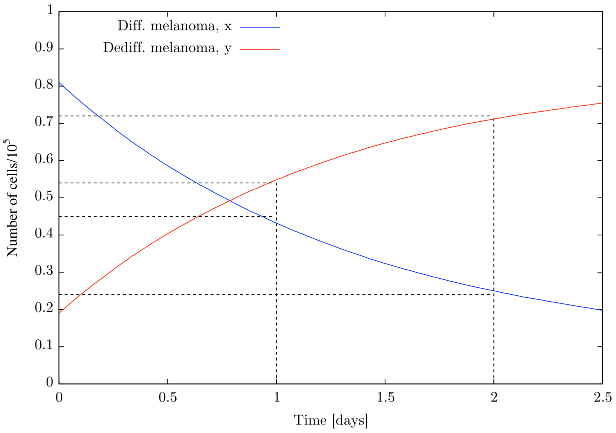

FIGURE 3. Switch in the in vitro experiments for inhibited cell division and constant concentration of TNF-α. The dashed lines indicate the experimental data.

rate depending on the amount of cancer cells present, $b(z_x, x) = b(z_y, y) = 2$ and produce one TNF-α molecule when they divide, $\ell_w^{\text{prod}}(z_x, x) = \ell_w^{\text{prod}}(z_y, y) = 1$. Furthermore, we assume that 4.5 cancer cells can be killed per hour (including indirect mechanisms), $t(z_x, x) = t(z_y, y) = 108$. The rate of death for the T-cell population is chosen as $d(z_x) = d(z_y) = 0.12$. These parameters are chosen such that the qualitative behavior of the tumor was recovered. We choose the same parameters for the second T-cell type as for the first one because there are no data concerning the second T-cell type.

- *Choice of starting values and the scale K:* We set $K = 10^5$, the initial value for the differentiated melanoma cell population to 1 and to 0 for the population of dedifferentiated melanoma cells. The ratio of differentiated and dedifferentiated cells is not known for small tumours which do not result from cell transfer of cells of in vitro cell lines. The initial value of the T-cell population is set to 0.02. We assume that the T-cells appear directly in the tumour, i.e. the migration phase into the tumour is not modelled.

To sum up, biological rates (per day) and initial conditions (in 100 000 cells) are:

$$\begin{array}{llll}
b(x) = 0.12 & b(y) = 0.12 & b(z_x, x) = 2 & \ell_w^{\text{prod}}(z_x, x) = 1 \\
d(x) = 0.02 & d(y) = 0.02 & t(z_x, x) = 108 & \ell_w^{\text{kill}}(z_x, x) = 0 \\
c(x, x) = 5 \cdot 10^{-5} & c(x, y) = 2 \cdot 10^{-5} & d(z_x) = 0.12 & d(w) = 0.2 \\
c(y, x) = 2 \cdot 10^{-5} & c(y, y) = 5 \cdot 10^{-5} & & s_w(x, y) = 0.33 \\
s(x, y) = 0.0008 & s(y, x) = 0.065 & & \\
\mathfrak{n}_x(0) = 1 & \mathfrak{n}_y(0) = 0 & \mathfrak{n}_{z_x}(0) = 0.02 & K = 10^5
\end{array} \tag{2.9}$$

The additional parameters in the case where a second T-cell is used are:

$$\begin{array}{lll}
t(z_y, y) = 108 & \ell_w^{\text{prod}}(z_y, y) = 1 & d(z_y) = 0.12 \\
b(z_y, y) = 2 & \ell_w^{\text{kill}}(z_y, y) = 0 & \mathfrak{n}_{z_y}(0) = 0.02
\end{array} \tag{2.10}$$

3. ARRIVAL OF A MUTANT

In this section, we give a more detailed explanation of our generalization of the Polymorphic Evolution Sequence (PES) in the case of fast switches in the phenotypic space. Recall that we consider the case of rare mutations in large populations on a timescale such that a population reaches equilibrium before a new mutant appears:

$$\forall V > 0, \qquad \exp(-VK) \ll \mu_g^K \ll \frac{1}{K \log K}, \qquad \text{as } K \to \infty. \tag{3.1}$$

The long-term behaviour of the standard models for adaptive dynamics is described in this limit by the Trait Substitution Sequence (TSS) (see [4, 5]) which is a Markov jump process on the trait space. A natural extension to the case where traits can coexist is the PES [6].

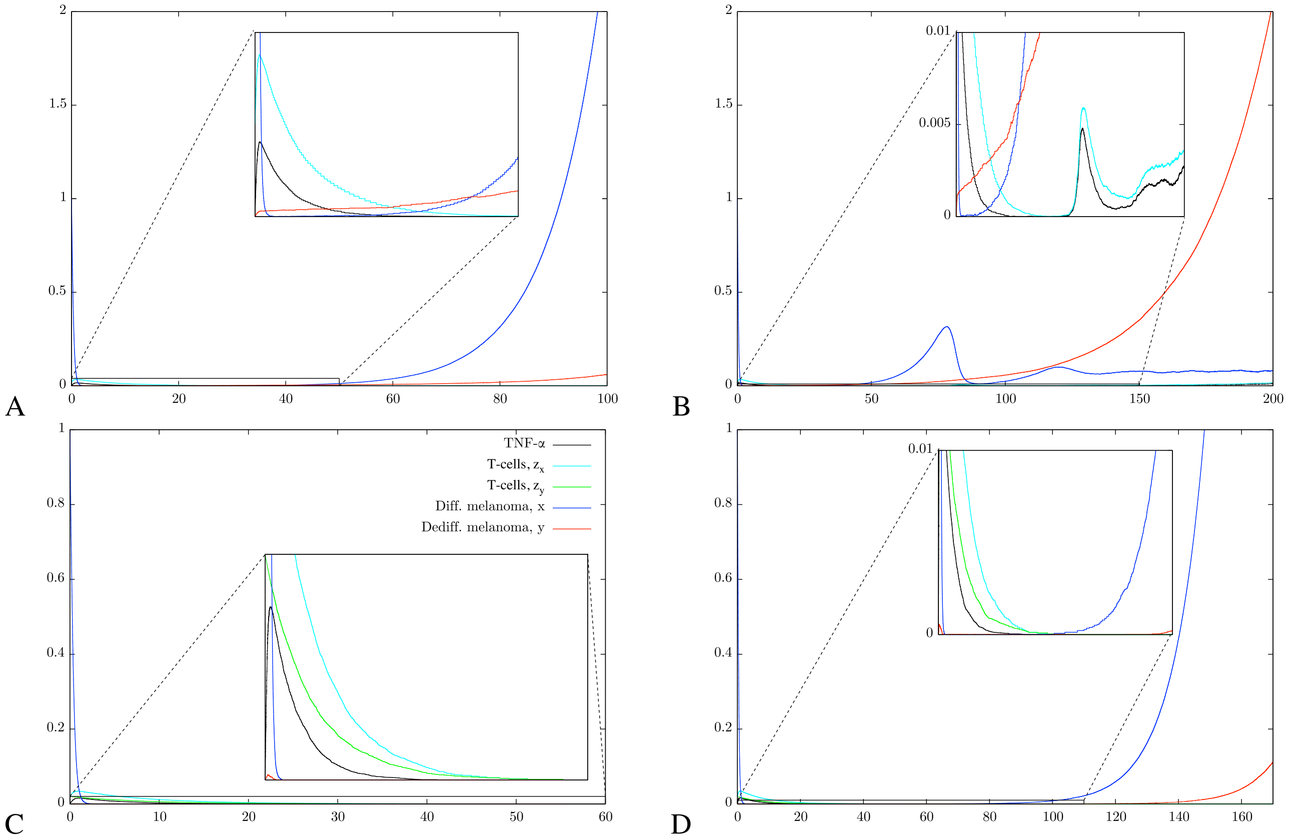

FIGURE 4. Simulations for biological parameters. Therapy with one T-cell type: (A) differentiated relapse (T-cells z_x die out), (B) dedifferentiated relapse (T-cells z_x survive), Therapy with two T-cell types: (C) cure, (D) differentiated relapse (both T-cell types die out).

Let us roughly describe how one gets from the individual-based model to this Markov jump process in the usual case, i.e. where the large population limit is a competitive Lotka-Volterra system with a unique stable[1] fixed point, see [5]. The main task is to study the invasion of a mutant that has just appeared in a population close to a stable fixed point. The invasion can be divided into three steps. First, as long as the mutant population size is smaller than $K\epsilon$ for a fixed small $\epsilon > 0$, the resident population stays close to its equilibrium. Therefore the mutant population can be approximated by a binary branching process. Second, once the mutant population reaches the level $K\epsilon$, for large K, the whole system is close to the solution of the corresponding deterministic system (this is a consequence of Proposition 1). Since the system has a unique stable fixed point, $\bar{\mathfrak{n}}$, the solution reaches a ϵ-neighbourhood of $\bar{\mathfrak{n}}$ in finite time. Finally, we can approximate the subpopulations which have a zero coordinate in $\bar{\mathfrak{n}}$ by sub-critical branching processes. The time of the first and third step are proportional to $\log(K)$, whereas the time of the second step is bounded. The second inequality in (3.1) guarantees that, with high probability, the three steps of invasion are completed before a new mutation occurs.

Invasion fitness. We recall that, given a population in a stable equilibrium that populates a certain set of traits, say $M \subset \mathcal{X}$, the invasion fitness $f(x, M)$ is the growth rate of a population consisting of a single individual with trait $x \notin M$ in the presence of the equilibrium population $\bar{\mathfrak{n}}$ on M. In the classical case it is simply given by

$$f(x, M) = b(x) - d(x) - \sum_{y \in M} c(x, y)\bar{\mathfrak{n}}_y. \tag{3.2}$$

Positive $f(x, M)$ implies that a mutant appearing with phenotype x from the equilibrium population on M has a positive probability (uniformly in K) to grow to a population of size of order K; negative invasion fitness implies that such a mutant population will die out with probability tending to one (as $K \to \infty$) before this happens.

[1]By stable fixed point we mean that the eigenvalues of the Jacobian matrix of the system at the fixed point have all strictly negative real parts.

We now generalize this notion to the case when fast phenotypic switches are present, in the case of pure tumour evolutions, i.e. we ignore the T-cells and the TNF-α.

Let us consider an initial population of genotype g (associated with ℓ different phenotypes $p_1, \ldots, p_\ell$) which is able to mutate at rate μ_g^K to another genotype g', associated with k different phenotypes $p'_1, \ldots, p'_k$. The assumption (3.1) ensures that no mutation occurs during the equilibration phase in the phenotypic space.

Consider as initial condition $\mathfrak{n}(0) = (\mathfrak{n}_{(g,p_1)}(0), \ldots, \mathfrak{n}_{(g,p_\ell)}(0))$ a stable fixed point, $\bar{\mathfrak{n}}$, of the following system:

$$\dot{\mathfrak{n}}_{(g,p_i)} = \mathfrak{n}_{(g,p_i)} \left(b_i - d_i - \sum_{j=1}^{\ell} c_{ij} \mathfrak{n}_{(g,p_j)} - \sum_{j=1}^{\ell} s_{ij} \right) + \sum_{j=1}^{\ell} s_{ji} \mathfrak{n}_{(g,p_j)}, \qquad i = 1, \ldots, \ell. \tag{3.3}$$

We write for simplicity $b_i = b(p_i)$, $d_i = d(p_i)$, $c_{ij} = c(p_i, p_j)$, $s_{ij} = s_g(p_i, p_j)$, and later $b'_i = b(p'_i)$, $d'_i = d(p'_i)$, $c'_{ij} = c(p'_i, p'_j)$, $\tilde{c}_{ij} = c(p_i, p'_j)$, $s'_{ij} = s_{g'}(p'_i, p'_j)$.

We want to analyse whether a single mutant appearing with a new genotype g' (and one of its possible phenotypes p'_i), has a positive probability to give rise to a population of size of order K. Using the same arguments as Champagnat et al. [5, 6], it is easy to show that as long as the mutant population has less than ϵK individuals (with $\epsilon \ll 1$), the mutant population $(g', p'_1), \ldots, (g', p'_k)$ is well approximated by a k-type branching process, where each individual undergoes binary branching, death, or switch to another phenotype with the following rates:

$$\left.\begin{array}{lll} p'_i \to p'_i p'_i & \text{with rate} & b'_i \\ p'_i \to \varnothing & \text{with rate} & d'_i + \sum_{l=1}^{\ell} \tilde{c}_{il} \bar{\mathfrak{n}}_l \\ p'_i \to p'_j & \text{with rate} & s'_{ij} \end{array}\right\} \quad \text{for } i, j \in \{1, \ldots, k\}. \tag{3.4}$$

where $(\bar{\mathfrak{n}}_1, \ldots, \bar{\mathfrak{n}}_\ell)$ denotes the fixed point of (3.3). We will assume that the switch rates s'_{ij} are the transition rates of an irreducible Markov chain on $\{1, \ldots, k\}$. The simplest example is the case where $s'_{ij} > 0$, for all $i, j \in \{1, \ldots, k\}$.

Multi-type branching processes have been analysed by Kesten and Stigum [7, 8, 9] and Athreya and Ney [10]. Their behaviour are classified in terms of the matrix A, given by

$$A = \begin{pmatrix} f_1 & s'_{12} & \ldots & s'_{1k} \\ s'_{21} & f_2 & & \vdots \\ \vdots & & \ddots & \\ s'_{k1} & \ldots & & f_k \end{pmatrix}, \tag{3.5}$$

where

$$f_i := b'_i - d'_i - \sum_{l=1}^{\ell} \tilde{c}_{il} \cdot \bar{\mathfrak{n}}_l - \sum_{j=1}^{k} s'_{ij}, \quad i = 1, \ldots, k. \tag{3.6}$$

Note that f_i *would be* the invasion fitness of phenotype i *if* there was no switch back from the other phenotypes to i (or if all switched cells would be killed). It is well-known that the multi-type process is super-critical, if and only if the largest eigenvalue, $\lambda_1 = \lambda_1(A)$, of the matrix A is strictly positive, meaning that if $\lambda_1 > 0$, the mutant population will grow (with rate λ_1) to any desired population size before dying out. Thus $\lambda_1(A)$ is the appropriate generalization of the invasion fitness of a genotype:

$$F(g', g) := \lambda_1(A). \tag{3.7}$$

This notion can easily be generalized to the case when the initial condition is the equilibrium population of several coexisting genotypes. Note that this notion of invasion fitness of course reduces to the standard one of [5] if there is only one mutant phenotype. This settles the first step of our analysis, which is the invasion of the mutant.

Towards a generalized PES. In fact, one can say more about how the mutant population grows. Write $Z_j^{(i)}(t)$ for the number of individuals of phenotype p_j existing at time t for this branching process when the first mutant is of phenotype p_i. Then, for $i, j \leq k$,

$$\mathbb{E}(Z_j^{(i)}(t)) = [M(t)]_{i,j} \tag{3.8}$$

where $M(t)$ is the $k \times k$-matrix

$$M(t) = \exp(At). \tag{3.9}$$

Assume that the largest eigenvalue $\lambda_1(A)$ is simple and strictly positive. Let v and u be the left and right eigenvectors of A associated to λ_1, normalized such that $u \cdot v = 1$ and $u \cdot 1 = 1$. The extinction probability vector $q = (q_1, \dots, q_k)$ where $q_i = \mathbb{P}(Z^{(i)}(t) = 0 \text{ for some } t)$ is the unique solution of the system of equations:

$$d'_i + \sum_{l=1}^{\ell} \tilde{c}_{il}\bar{\mathfrak{n}}_l + b'_i q_i^2 + \sum_{j=1}^{k} s'_{ij} q_j = q_i \left(d'_i + \sum_{l=1}^{\ell} \tilde{c}_{il}\bar{\mathfrak{n}}_l + b'_i + \sum_{j=1}^{k} s'_{ij} \right), \qquad i = 1, \dots, k \tag{3.10}$$

which has in general no analytical solution. Then the following limit theorem holds [7, 10]:

$$\lim_{t\to\infty} \left(Z_1^{(i)}(t), \dots, Z_k^{(i)}(t) \right) e^{-\lambda'_1 t} = W_i \cdot (v_1, \dots, v_k) \quad \text{a.s.} \tag{3.11}$$

where $(W_i)_{i=1,\dots,k}$ is random vector with non-negative entries such that

$$\mathbb{P}(W_i = 0) = q_i \quad \text{and} \quad \mathbb{E}(W_i) = u_i. \tag{3.12}$$

In particular, conditionally on survival, the phenotypic distribution of the mutant populations converges almost surely to a deterministic quantity, which moreover does not depend on the phenotype of the initial mutant, namely

$$\lim_{t\to\infty} \frac{Z_{j'}^{(i)}(t)}{\sum_{j=1}^{k} Z_j^{(i)}(t)} = \frac{v_{j'}}{\sum_{j=1}^{k} v_j}, \quad \forall i, j' = 1, \dots, k. \tag{3.13}$$

For us, this implies the important fact that the phenotypic structure of the mutant population once it reaches the level $\varepsilon K > 0$ is almost deterministic. Then, conditionally on survival, (3.11) implies that the time $\tau_{\varepsilon K}$ until the total mutant population reached εK is of order $\log(K)$. Moreover, the proportions of the k types of phenotypes converge to deterministic quantities given above,

$$\frac{1}{K}(Z_1(\tau_{\varepsilon K}), \dots, Z_k(\tau_{\varepsilon K})) \to \left(\frac{\varepsilon v_1}{\sum_{j=1}^{k} v_j}, \dots, \frac{\varepsilon v_k}{\sum_{j=1}^{k} v_j} \right), \quad \text{in distribution, as } K \to \infty. \tag{3.14}$$

Once the mutant population has reached the level εK, the behaviour of the process can be approximated by the solution of the deterministic system:

$$\begin{aligned} \dot{\mathfrak{n}}_{(g,p_i)} &= \mathfrak{n}_{(g,p_i)} \left(b_i - d_i - \sum_{j=1}^{\ell} s_{ij} - \sum_{j=1}^{\ell} c_{ij}\mathfrak{n}_{(g,p_j)} - \sum_{j=1}^{k} \tilde{c}_{ij}\mathfrak{n}_{(g',p'_j)} \right) + \sum_{j=1}^{\ell} s_{ji}\mathfrak{n}_{(g,p_j)}, \\ \dot{\mathfrak{n}}_{(g',p'_i)} &= \mathfrak{n}_{(g',p'_i)} \left(b'_i - d'_i - \sum_{j=1}^{k} s'_{ij} - \sum_{j=1}^{k} c'_{ij}\mathfrak{n}_{(g',p'_j)} - \sum_{j=1}^{\ell} \tilde{c}_{ij}\mathfrak{n}_{(g,p_j)} \right) + \sum_{j=1}^{k} s'_{ji}\mathfrak{n}_{(g',p'_j)}, \end{aligned} \tag{3.15}$$

(where i runs from 1 to ℓ in the first line and from 1 to k in the second one) with initial conditions in a small neighbourhood of

$$(\mathfrak{n}_{(g,p_1)}(0), \dots, \mathfrak{n}_{(g,p_\ell)}(0), \mathfrak{n}_{(g',p'_1)}(0), \dots, \mathfrak{n}_{(g',p'_k)}(0)) = \left(\bar{\mathfrak{n}}_1, \dots, \bar{\mathfrak{n}}_\ell, \frac{\varepsilon v_1}{\sum_{j=1}^{k} v_j}, \dots, \frac{\varepsilon v_k}{\sum_{j=1}^{k} v_j} \right). \tag{3.16}$$

If the system (3.15) is such that a neighbourhood of (3.16) is attracted to the same stable fixed point, we are in the same situation as in Champagnat and Méléard [6] and get the generalization of the Polymorphic Evolution Sequence on the genotypic trait space.

The characterization of the asymptotic behaviour of the system (3.15) is needed to describe the final state of our stochastic process. This is in general a very difficult and complex problem, which is not doable analytically and requires numerical analysis.

On Figure 5 (A) of the main part is an example with the following parameters:

$$\begin{array}{llll} b_0 = 5 & b_1 = 6 & b_2 = 6 & s_{12} = 0.1 \\ d_0 = 0 & d_1 = 0 & d_2 = 0 & s_{21} = 2 \\ c_{00} = 1 & c_{10} = 1 & c_{20} = 1 & \\ c_{01} = 1 & c_{11} = 1 & c_{21} = 0 & \\ c_{02} = 0 & c_{12} = 0 & c_{22} = 1 & \\ \mathfrak{n}_{(g,p)}(0) = 5 & \mathfrak{n}_{(g',p'_1)}(0) = 0 & \mathfrak{n}_{(g',p'_2)}(0) = 1 & K = 200 \end{array} \tag{3.17}$$

In this case, p_2' can switch to p_1' but the back-switch is very weak, and we have $f_2 < 0$ and $f_1 > 0$ according to definition (3.6). The global fitness of the genotype g' is positive and close to f_1. The coexistence of the two phenotypes depends on the cross-competition c_{12} and c_{21}.

Figure 5 (B) of the main part shows an example of the case discussed above with the following parameters:

$$\begin{array}{llll} b_0 = 5 & b_1 = 6 & b_2 = 6 & s_{12} = 2 \\ d_0 = 0 & d_1 = 0 & d_2 = 0 & s_{21} = 2 \\ c_{00} = 1 & c_{10} = 1 & c_{20} = 1 & \\ c_{01} = 1 & c_{11} = 1 & c_{21} = 0 & \\ c_{02} = 0 & c_{12} = 0 & c_{22} = 1 & \\ \mathfrak{n}_{(g,p)}(0) = 5 & \mathfrak{n}_{(g',p_1')}(0) = 1 & \mathfrak{n}_{(g',p_2')}(0) = 0 & K = 200 \end{array} \tag{3.18}$$

For these parameters f_1 and f_2 as defined in (3.6) are negative, but, due to the cooperation of the two phenotypes, the fitness of the genotype is positive and it invades with positive probability as indicated by the definition (3.7). Moreover, both phenotypes appear on a macroscopic level.

4. Influence of birth-reducing competition on mutation events

Let us discuss in more detail how the birth-reducing competition can have a crucial effect on the mutation timescale. For the sake of simplicity we consider an example where the switching rates are set to 0. Consider a melanoma population (g, p) which is able to mutate to a fitter type of melanoma (g', p'). We allow for one T-cell population attacking the resident melanoma population since this is the simplest scenario where the effect of therapy in this context can be explained. As the presence of TNF-α only influences the switch between phenotypes, it does not play any role in this example and we can set the corresponding parameters (ℓ_w and $d(w)$) to zero without loss of generality. If $\mu_g^K \to 0$ as $K \to \infty$ the limiting deterministic system is given by:

$$\begin{aligned} \dot{\mathfrak{n}}_{(g,p)} &= \mathfrak{n}_{(g,p)} \left(b(p) - d(p) - c_b(p,p)\mathfrak{n}_{(g,p)} - c_b(p,p')\mathfrak{n}_{(g',p')} - t(z,p)\mathfrak{n}_z \right) \\ \dot{\mathfrak{n}}_{(g',p')} &= \mathfrak{n}_{(g',p')} \left(b(p') - d(p') - c_b(p',p')\mathfrak{n}_{(g',p')} - c_b(p',p)\mathfrak{n}_{(g,p)} \right) \\ \dot{\mathfrak{n}}_z &= \mathfrak{n}_z (b(z,p)\mathfrak{n}_{(g,p)} - d(z)) \end{aligned} \tag{4.1}$$

Note that the mutation term does not appear in the deterministic system and that the difference between birth-reducing competition and usual competition is not visible. The effects we are looking for are intrinsically stochastic and happen on time-scales that diverge with K.

As explained in the main part of the paper, the interesting choice for the mutation rates is $K\mu_g^K \to \alpha > 0$ as $K \to \infty$.

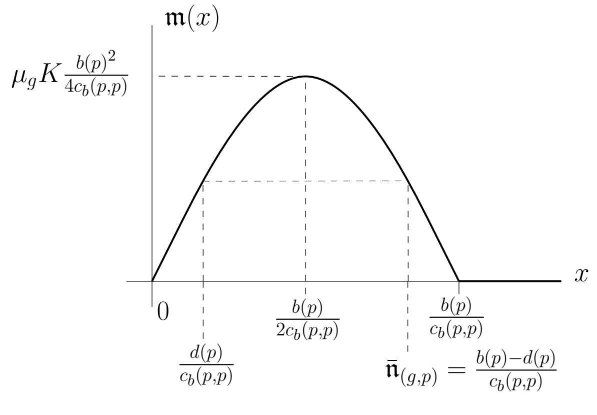

FIGURE 5. Shape of the initial total mutation rate of the population (g, p).

If the competition is only of birth-reducing type, then the total mutation rate of the population of type (g, p) at time t is

$$\mathfrak{m}(\nu_t^K(g,p)) := \mu_g^K \left\lfloor b(p) - c_b(p,p)\nu_t^K(g,p) \right\rfloor_+ \nu_t^K(g,p)K. \tag{4.2}$$

This is a positive and concave function of $\nu_t^K(g,p)$ on the interval $[0, b(p)/c_b(p,p)]$, see Figure 5. If the population is at equilibrium (without or before therapy), meaning $\nu_t^K(g,p) = \bar{\mathfrak{n}}_{(g,p)} = (b(p) - d(p))/c_b(p,p)$, then the time until a mutation occurs is approximately exponentially distributed with parameter equal to

$$\mu_g^K K \cdot \left(b(p) - c_b(p,p)\bar{\mathfrak{n}}_{(g,p)} \right) \bar{\mathfrak{n}}_{(g,p)} = \mu_g^K K \cdot d(p)\bar{\mathfrak{n}}_{(g,p)}. \tag{4.3}$$

If $d(p)$ is smaller than $b(p)/2$, then $\bar{\mathfrak{n}}_{(g,p)}$ is bigger than $b(p)/2c_b(p,p)$ and $\mathfrak{m}(\bar{\mathfrak{n}}_{(g,p)})$ is not maximal. Smaller populations, more precisely in between $d(p)/c_b(p,p)$ and $\bar{\mathfrak{n}}_{(g,p)}$, have a higher total mutation rate.

This situation can happen during a T-cell therapy. Indeed, the introduction of T-cells in the system lowers the average population of melanoma (usually by making it undergo a damped cycle), and there exist parameters such that the mutation rate of (g,p) is larger during the treatment, for the reason explained above. Figure 6 of

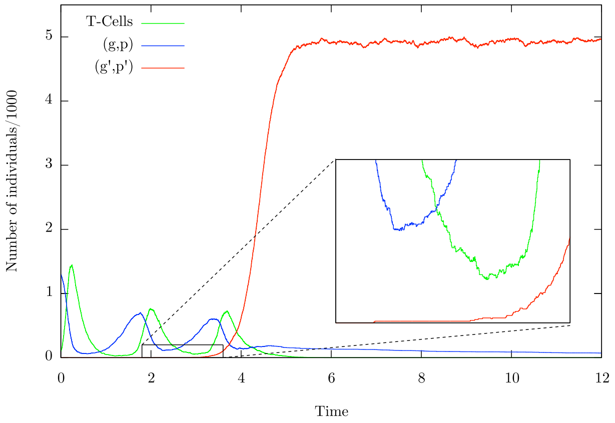

FIGURE 6. Example of earlier mutation induced by therapy, with parameters given by (4.4). Starting with a population at its equilibrium with therapy.

the main part illustrates this situation with the following parameters:

$$\begin{array}{llll} b(p)=4 & b(p')=6 & b(z,p)=20 & m((g,p),(g',p'))=1 \\ d(p)=0.1 & d(p')=1 & t(z,p)=10 & \mu_g=10^{-3} \\ c_b(p,p)=3 & c_b(p',p)=0.8 & d(z)=6 & K=10^3 \\ c_b(p,p')=0.8 & c_b(p',p')=1 & & \\ \mathfrak{n}_{(g,p)}(0)=1.3 & \mathfrak{n}_{(g',p')}(0)=0 & \mathfrak{n}_z(0)=0 \text{ or } 0.1 & \end{array} \tag{4.4}$$

Figure 6 shows another realization of the case with T-cell therapy, with average mutation time smaller than in the equilibrium case, and a mutation happening in the second minimum of the melanoma population.

Note that this example provides an interesting situation of interplay between therapy and mutation. By lowering the melanoma population, the T-cell therapy actually increases the probability for it to mutate to a potentially fitter and pathogenic genotype, which is not affected by the T-cells.

5. SIMULATIONS

We use the following notations: let $\mathcal{D}$ be some discrete set and X a $\mathcal{D}$-valued random variable, then X sampled according to the weights $\{w(i), i \in \mathcal{D}\}$ means that $\mathbb{P}(X = i) = w(i)/\sum_{i\in\mathcal{D}} w(i)$.

```
Data: initial conditions: ν_0^K ∈ M^K(X), number of iterations: N_Iterations, parameters described in Section 1
T_0 ← 0, ν_{T_0}^K ← ν_0^K, k ← 0
while k ≤ N_Iterations do
    for x ∈ Supp(ν_{T_k}^K) do
        if x = (g,p) ∈ G × P then
            B(x) ← K ν_{T_k}^K(g,p) ⌊ b(p) − Σ_{(g̃,p̃)∈Supp(ν_{T_k}^K)} c_b(p,p̃) ν_{T_k}^K(g̃,p̃) ⌋_+
            D(x) ← K ν_{T_k}^K(g,p) ( d(p) + ⌊ b(p) − Σ_{(g̃,p̃)∈Supp(ν_{T_k}^K)} c_b(p,p̃) ν_{T_k}^K(g̃,p̃) ⌋_−
                        + Σ_{(g̃,p̃)∈Supp(ν_{T_k}^K)} c(p,p̃) ν_{T_k}^K(g̃,p̃) ),
            T(x) ← K ν_{T_k}^K(g,p) Σ_{z∈Supp(ν_{T_k}^K)} t(z,p) ν_{T_k}^K(z),
            S(x) ← K ν_{T_k}^K(g,p) Σ_{p̃∈P} ( s^g(p,p̃) + Σ_{w∈Supp(ν_{T_k}^K)} s_w^g(p,p̃) ν_{T_k}^K(w) ),
            P(x) ← 0,
        if x = z ∈ Z then
            B(x) ← K ν_{T_k}^K(z) b(z),   D(x) ← K ν_{T_k}^K(z) d(z),   T(x) ← 0,   S(x) ← 0
            P(x) ← K ν_{T_k}^K(z) Σ_{(g,p)∈Supp(ν_{T_k}^K)} b(z,p) ν_{T_k}^K(g,p),
        if x = w ∈ W then
            B(x) ← 0,   D(x) ← K ν_{T_k}^K(w) d(w),   T(x) ← 0,   S(x) ← 0,   P(x) ← 0,
        TotalTraitRate(x) ← B(x) + D(x) + T(x) + P(x) + S(x)
    TotalEventRate ← Σ_{x∈Supp(ν_{T_k}^K)} TotalTraitRate(x)
    Sample t ~ Exp(TotalEventRate)
    T_{k+1} ← T_k + t
    Sample x_chosen ∈ X according to the weights {TotalTraitRate(x), x ∈ Supp(ν_{T_k}^K)}.
    Sample the event E ∈ {Birth, Death, Therapy, Production, Switch} according to the weights
    {B(x_chosen), D(x_chosen), T(x_chosen), P(x_chosen), S(x_chosen)}
    case E = Birth
        if x_chosen = (g,p) ∈ G × P then
            Sample B ∈ {No Mutation, Mutation} according to {1 − μ_g, μ_g}
            case B = No Mutation
                ν_{T_{k+1}}^K ← ν_{T_k}^K + (1/K) δ_{x_chosen}
            case B = Mutation
                Sample (g̃,p̃) according to m((g,p),(g̃,p̃))
                ν_{T_{k+1}}^K ← ν_{T_k}^K + (1/K) δ_{(g̃,p̃)}
        else
            ν_{T_{k+1}}^K ← ν_{T_k}^K + (1/K) δ_{x_chosen}
    case E = Death
        ν_{T_{k+1}}^K ← ν_{T_k}^K − (1/K) δ_{x_chosen}
    case E = Therapy
        Note that x_chosen = (g,p) for some (g,p) ∈ G × P in this case.
        Sample z ∈ Z according to the weights {t(z,p) ν_{T_k}^K(z), z ∈ Supp(ν_{T_k}^K) ∩ Z}
        ν_{T_{k+1}}^K ← ν_{T_k}^K − (1/K) δ_{(g,p)} + Σ_{w∈W} ℓ_w^kill(z,p) (1/K) δ_w
    case E = Production
        Sample (g,p) ∈ Supp(ν_{T_k}^K) according to the weights {b(x_chosen,p) ν_{T_k}^K(g,p), (g,p) ∈ Supp(ν_{T_k}^K)}
        ν_{T_{k+1}}^K ← ν_{T_k}^K + (1/K) δ_{x_chosen} + Σ_{w∈W} ℓ_w^prod(x_chosen,p) (1/K) δ_w
    case E = Switch
        Note that x_chosen = (g,p) for some (g,p) ∈ G × P in this case.
        Sample p̃ ∈ P according to { s^g(p,p̃) + Σ_{w∈Supp(ν_{T_k}^K)} s_w^g(p,p̃) ν_{T_k}^K(w), p̃ ∈ P }
        ν_{T_{k+1}}^K ← ν_{T_k}^K − (1/K) δ_{(g,p)} + (1/K) δ_{(g,p̃)}
    k = k + 1
```

Algorithm 1: Pseudo-code of the Gillespie algorithm used for generating the figures in this article.

The depth diagram of the algorithm we used to generate the simulations in this article is given in Figure 7. The pseudo-code is given in Algorithm (1).

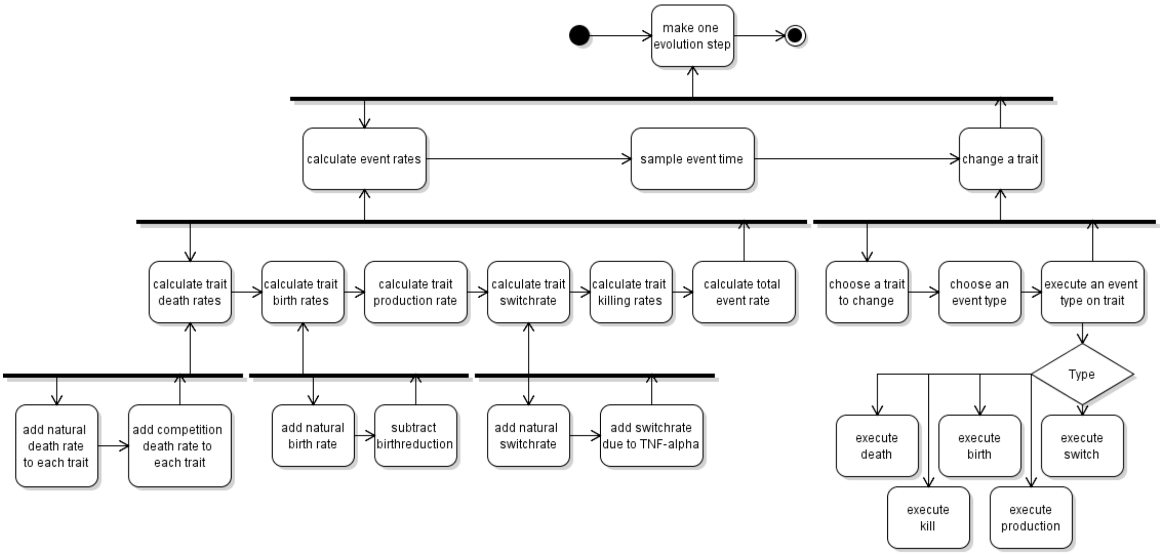

FIGURE 7. Depth diagram of the program.

REFERENCES

[1] Ethier, S. N. & Kurtz, T. G. *Markov processes*, (John Wiley & Sons, Inc., 1986).

[2] Fournier, N. & Méléard, S. A microscopic probabilistic description of a locally regulated population and macroscopic approximations. *Ann. Appl. Probab.* **14,** 1880–1919 (2004).

[3] Landsberg, J. et al. Melanomas resist t-cell therapy through inflammation-induced reversible dedifferentiation. *Nature* **490,** 412–416 (2012).

[4] Metz, J. A. J., Geritz, S. A. H., Meszéna, G., Jacobs, F. J. A. & van Heerwaarden, J. S. Adaptive dynamics, a geometrical study of the consequences of nearly faithful reproduction in *Stochastic And Spatial Structures Of Dynamical Systems*, (eds van Strien, S. J. & Verduyn Lunel, S. M.), 183–231 (Konink. Nederl. Akad. Wetensch. Verh. Afd. Natuurk. Eerste Reeks, 1995).

[5] Champagnat, N. A microscopic interpretation for adaptive dynamics trait substitution sequence models. *Stoch. Process. Their Appl.* **116,** 1127–1160 (2006).

[6] Champagnat, N. & Méléard, S. Polymorphic evolution sequence and evolutionary branching. *Probab. Theory Relat. Fields* **151,** 45–94 (2011).

[7] Kesten, H. & Stigum, B. P. A limit theorem for multidimensional Galton-Watson processes. *Ann. Math. Stat.* **37,** 1211–1223 (1966).

[8] Kesten, H. & Stigum, B. P. Additional limit theorems for indecomposable multidimensional Galton-Watson processes. *Ann. Math. Stat.* **37,** 1463–1481 (1966).

[9] Kesten, H. & Stigum, B. P. Limit theorems for decomposable multi-dimensional Galton-Watson processes. *J. Math. Anal. Appl.* **17,** 309–338 (1967).

[10] Athreya, K. B. & Ney, P. E. *Branching Processes* (Springer, 1972).
